# Longitudinal monitoring of immunoglobulin A glycosylation during pregnancy by simultaneous MALDI-FTICR-MS analysis of *N*- and *O*-glycopeptides

**DOI:** 10.1038/srep27955

**Published:** 2016-06-15

**Authors:** Albert Bondt, Simone Nicolardi, Bas C. Jansen, Kathrin Stavenhagen, Dennis Blank, Guinevere S. M. Kammeijer, Radoslaw P. Kozak, Daryl L. Fernandes, Paul J. Hensbergen, Johanna M. W. Hazes, Yuri E. M. van der Burgt, Radboud J. E. M. Dolhain, Manfred Wuhrer

**Affiliations:** 1 Department of Rheumatology, Erasmus University Medical Center, Rotterdam, The Netherlands; 2 Center for Proteomics and Metabolomics, Leiden University Medical Center, Leiden, The Netherlands; 3 Division of BioAnalytical Chemistry, VU University Amsterdam, The Netherlands; 4 Ludger, Culham Science Centre, Oxfordshire OX14 3EB, UK

## Abstract

Immunoglobulin A (IgA) is a glycoprotein of which altered glycosylation has been associated with several pathologies. Conventional methods for IgA *N*- and *O*-glycosylation analysis are tedious, thus limiting such analyses to small sample sizes. Here we present a high-throughput strategy for the simultaneous analysis of serum-derived IgA1 *N*- and *O*-glycopeptides using matrix-assisted laser/desorption ionisation Fourier transform ion cyclotron resonance (MALDI-FTICR) mass spectrometry (MS). Six non-fucosylated diantennary complex type glycoforms were detected on the Asn144-containing glycopeptide. Thirteen distinct glycoforms were identified for the Asn340-containing tailpiece glycopeptide, mainly of the diantennary complex type, and low amounts of triantennary glycoforms. Simultaneously with these *N*-glycopeptides, 53 compositional glycoforms of the hinge region *O*-glycopeptide were profiled in a single high resolution MALDI-FTICR spectrum. Since many pregnancy associated changes have been recognized for immunoglobulin G, we sought to demonstrate the clinical applicability of this method in a cohort of 29 pregnant women, from whom samples were collected at three time points during pregnancy and three time points after delivery. Pregnancy associated changes of *N*-glycan bisection were different for IgA1 as compared to IgG-Fc described earlier. We foresee further applications of the developed method for larger patient cohorts to study IgA *N*- and *O*-glycosylation changes in pathologies.

Human antibodies consist of two light chains (which can be immunoglobulin kappa or lambda) and two heavy chains (either immunoglobulin alpha, delta, epsilon, gamma or mu). The heavy chains determine the antibody isotypes (IgA, IgD, IgE, IgG or IgM, respectively). For immunoglobulin A two subclasses exist, namely IgA1 and IgA2, as defined by structure differences between the α1 or α2 heavy chains. IgA concentrations in the human circulation are about 2 mg/mL^1^, of which IgA1 accounts for ~90%. Both IgA1 and IgA2 are post-translationally modified by covalently connected *N*- and *O*-glycans (Fig. 1).

Amongst various single amino acid differences, IgA subclasses differ in the hinge region, where the primary sequence of IgA1 contains thirteen additional amino acids (aa) compared to IgA2 (see Supplementary Material S1). Previously, it was found that three *O*-glycosylation sites in this hinge region were constitutively occupied (Thr106, Thr109 and Ser113), whereas Ser111, Thr114 and Thr117 may or may not carry an *O*-glycan^2^,^3^. (In the current manuscript amino acid numbering is based on the constant part or the heavy chains only (UniProt entries: IGHA1/P01876, 353aa; IGHA2/P01877, 340aa), while the referred manuscripts use a numbering based on the traditionally expected total amino acid sequence. Due to the differences in numbering, these sites correspond to Thr225, Thr228, Ser232, Ser230, Thr233 and Thr236 in the referred papers, respectively.) These amino acids carry *O*-glycans of the core 1 type^2^,^3^,^4^,^5^,^6^,^7^. Such *O*-glycans consist of a single *N*-acetylgalactosamine (GalNAc) that can be extended with one galactose (resulting in the T antigen), and up to two sialic acids of which one can be attached to the GalNAc and one to the galactose.

IgA *N*-glycans are present at Asn144 and Asn131 in the C_H_2 domain of IgA1 and 2, respectively, and at Asn340 and Asn327 in the tail piece domain^8^. In addition, IgA2 contains two *N*-glycosylation sites at Asn47 in C_H_1 and at Asn205 in the C_H_2 domain^8^. The serum IgA *N*-glycans have been found to be of the diantennary complex type, with high levels of galactosylation and sialylation, and possible bisection and fucosylation^4^,^9^,^10^,^11^. More specifically, the C_H_2 derived glycan of human serum IgA1 was found to be diantennary without a fucose, in contrast to the fucosylated glycan on Asn340^11^. Others studied polymeric IgA from human colostrum or serum and confirmed the low levels of fucose on the C_H_2 glycan, although the glycans in general were smaller (mainly high mannose, non**- or mono**-galactosylated complex type)^12^.

It has been found that IgA plays an important role in several pathologies, e.g. IgA nephropathy (IgAN) and Henoch-Schönlein purpura (HSP). One of the main topics of research on IgAN is related to its *O*-glycosylation, which has been suggested to be involved in IgAN pathogenesis^13^,^14^. Moreover, it has been proposed as a marker for therapeutic response in IgAN treatment, with lowered levels of agalactosylated *O*-glycans as a marker of improvement^15^. Similarly, the galactose deficient IgA *O*-glycans have been associated with HSP^16^. This agalactosylated *O*-glycan is known as Tn**-antigen, and the sialylated form is known as sialyl-Tn-antigen. Rheumatoid arthritis also is associated with changed *O*-glycosylation of the IgA hinge region, namely lower levels of GalNAc compared to healthy individuals, while the number of galactoses remained the same^17^.

The role and underlying mechanisms of IgA1 *N*-glycosylation changes in disease are not yet unravelled. It has been suggested that glycosylation changes influence the binding of IgA to the Fc alpha receptor^4^, however this was not observed in a later study^18^. Notwithstanding, binding to FcαR could induce both pro-and anti-inflammatory processes^19^, and the role of the *N*-glycans in driving the process in either one of these directions is unknown. Furthermore, the *N*-glycosylation may affect binding to receptors responsible for IgA clearance, e.g. asialoglycoprotein receptor^20^,^21^. Finally, IgA has been found to bind to the glycan-binding DC-SIGN/SIGN-R1^22^, which for IgG (in mice) has been suggested to be involved in anti-inflammatory processes^23^. However, the physiological role of this is still under debate^24^.

Methods reported so far for the detection of IgA glycosylation involve lectin binding arrays^25^,^26^,^27^, mass spectrometry (MS) in combination with various chromatographic strategies^2^,^3^,^6^,^18^,^27^,^28^,^29^,^30^ and liquid chromatography (LC) with fluorescence detection^31^. These methodologies lack specificity (lectins) and are time-consuming (LC-MS). Sample throughput can be improved after reducing sample complexity using sialidases^3^, however at the expense of important information.

MS strategies based on matrix-assisted laser/desorption ionisation (MALDI) allow a high-throughput analysis of a complex mixture with the additional benefit of preventing carryover between samples. Most commonly, MALDI is coupled to time-of-flight (TOF) mass spectrometers. However, for simultaneous MALDI-MS analysis of *N*- and *O*-glycopeptides from polyclonal serum IgA high resolving powers (>50000) are pivotal. Moreover, sialic acids are instable in commercially available MALDI-TOF systems. Therefore, MALDI has hitherto hardly been applied for IgA *O*-glycopeptide analysis^32^. Fourier transform (FT) MS methods provide the highest resolving power and mass accuracy within the different mass spectrometers^33^. Previously, we have applied MALDI-FT- ion cyclotron resonance (ICR)-MS to map singly-charged proteins up to about 17000 Da and use the accurate mass differences for identification purposes^34^,^35^. These properties allow both the resolution of near isobaric species and the identification of possible post-translational modifications (PTMs). Another advantage of MALDI-FTICR-MS is the fact that the intermediate pressure in the MALDI source leads to a significant reduction of the sialic acid fragmentation^36^. Even though a MALDI approach will not allow for full discrimination of all macro- and microheterogeneity, it is suitable for high-throughput profiling in a clinical setting.

Therefore, in this study serum IgA glycopeptides were enriched with high specificity using a combination of purification techniques including a 96-well plate IgA affinity purification, trypsin digestion and two sequential HILIC solid-phase extractions. The enriched *N*- and *O*-glycopeptides were measured with isotopic resolution in an *m/z*-range from 3499 to 10000 using a 15T MALDI-FTICR mass spectrometer. This approach allowed for the first time the specific, high-throughput analysis of both *N*- and *O*-glycosylation of IgA1 by ultrahigh resolution MALDI-FTICR-MS. We observed 53 different compositions for the *O*-glycopeptide, 6 glycoforms for the Asn144 containing *N*-glycopeptide, and 13 glycoforms for the Asn340 containing glycopeptides. Confident assignment of the glycoforms was achieved making use of the high resolution and mass accuracy, together with fragment ion spectra. For immunoglobulin G it is known that many glycosylation changes occur with pregnancy, and therefore we applied our method on a pregnancy cohort (n = 29 individuals, 6 time points) to explore whether the same holds true for immunoglobulin A. The application of this method showed several site specific differences in *N*-glycosylation between pregnancy and the postpartum state. *O*-glycosylation showed some minor pregnancy associated alterations with respect to galactosylation and the ratio of galactoses per GalNAc.

## Results

### IgA capturing and digestion

We established a workflow for the high-throughput profiling of serum IgA1 *N*- and *O*-glycosylation by MALDI-FTICR-MS. IgA was affinity-captured from human serum in a 96-well format resulting in a highly enriched IgA sample (see Supplementary Table S1). The captured IgA was digested using trypsin after reduction and alkylation in order to generate specific glycopeptide fragments.

### MALDI-FTICR-MS profiling of IgA glycopeptides

**Figure 2:**
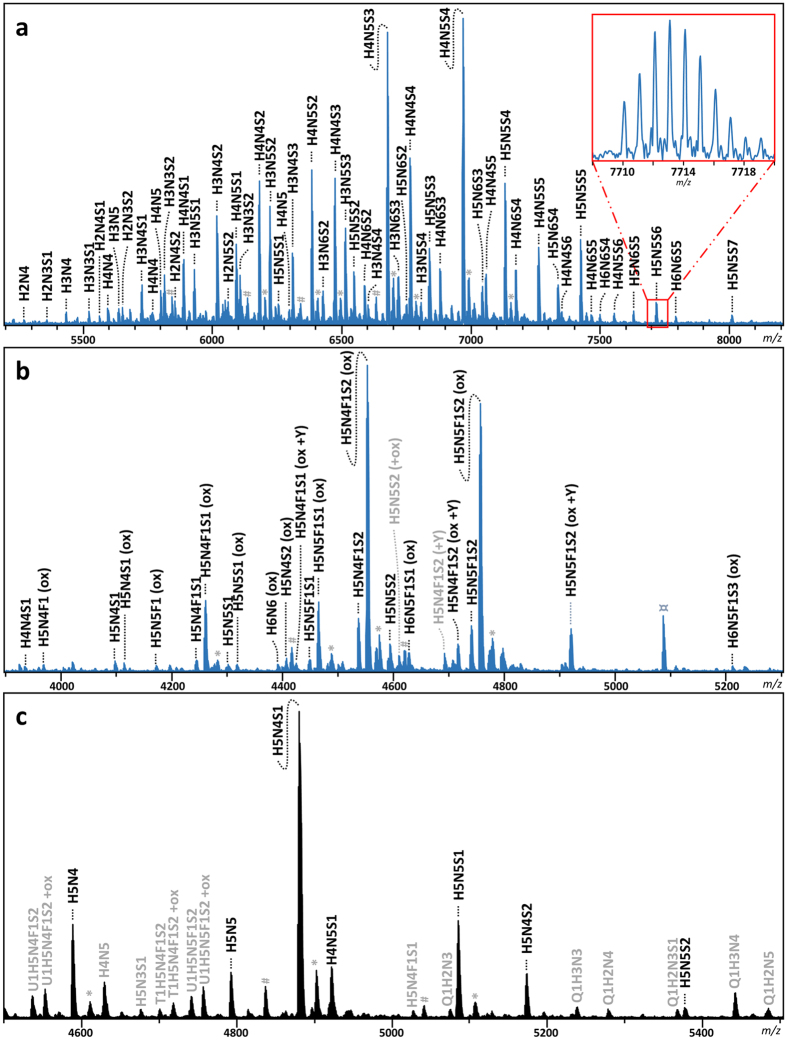
Typical spectra of IgA glycopeptides as obtained by MALDI-FTICR-MS after trypsin digestion and cotton HILIC SPE. Annotated peaks show the *O*-glycosylated hinge-region glycopeptide cluster (**a**) and the Asn340 glycopeptide cluster (non-truncated peptides are indicated by +Y; (**b**)) as obtained from a single mass spectrum from the 70% ACN HILIC step, and the Asn144 glycopeptide cluster (**c**) as obtained from a mass spectrum after 80% ACN HILIC. The assignment was supported by high mass accuracy, as well as LC-MS/MS of glycopeptides and MALDI-TOF-MS of released glycans. Glycopeptides that were not included in the final analysis after analyte curation are depicted in grey. Q1 = *O*-glycopeptide; T1 = non-truncated and U1 = truncated Asn340 containing glycopeptide; H = hexose; N = *N*-acetylhexosamine; F = fucose; S = *N*-acetylneuraminic acid; *sodium adduct; ^#^unidentified non-IgA related glycopeptide contaminant; ¤unidentified non-glycopeptide contaminant.

To be able to accurately detect all glycoforms present in the IgA glycopeptide mixture ultrahigh resolution MALDI-FTICR-MS measurements were performed using 4-chloro-α-cyanocinnamic acid matrix after HILIC enrichment of the IgA glycopeptides. HILIC enrichment was optimized to obtain the IgA1 hinge-region *O*-glycopeptides and the IgA1 Asn340 *N*-glycopeptides in one single spectrum after purification from 70% ACN, while the second IgA1 *N*-glycopeptide cluster (Asn144) was subsequently HILIC enriched from the flow-through starting with 80% ACN (see Methods section for the details) and obtained in a second spectrum (Fig. 2).

### Peak list generation

Manual inspection of the spectra showed no IgA2 specific glycopeptide clusters. For IgA1 several glycoforms were observed for the *N*-glycopeptides containing Asn144 (peptide moiety _127_LSLHRPALEDLLLGSEANLTCTLTGLR_153_) or containing Asn340 (peptide moiety _332_LAGKPTHVNVSVVMAEVDGTCY_353_), and for the hinge region *O*-glycopeptide (_89_HYTNPSQDVTVPCPVPSTPPTPSPSTPPTPSPSCCHPR_126_). In addition, some apparent mass deviations were observed, that could be explained by the loss of *C*-terminal tyrosine from the Asn340 containing glycopeptide, as was previously observed by others^18^,^30^, and by oxidation of the methionine in the Asn340 glycopeptides. Furthermore, we did observe some sodium adducts in the spectra, however these were generally minor and therefore not included in the analysis. In some spectra we observed contaminants and these were excluded from further analysis. The high resolving powers (>50000) that are characteristic of MALDI-FTICR-MS were crucial for simultaneous profiling of the *N*- and *O*-glycopeptides in a single spectrum. Moreover, the high mass accuracies (<2 ppm) provided by the system allowed confident assignment of the IgA glycopeptides (Supplementary Fig. S3).

**Figure 3:**
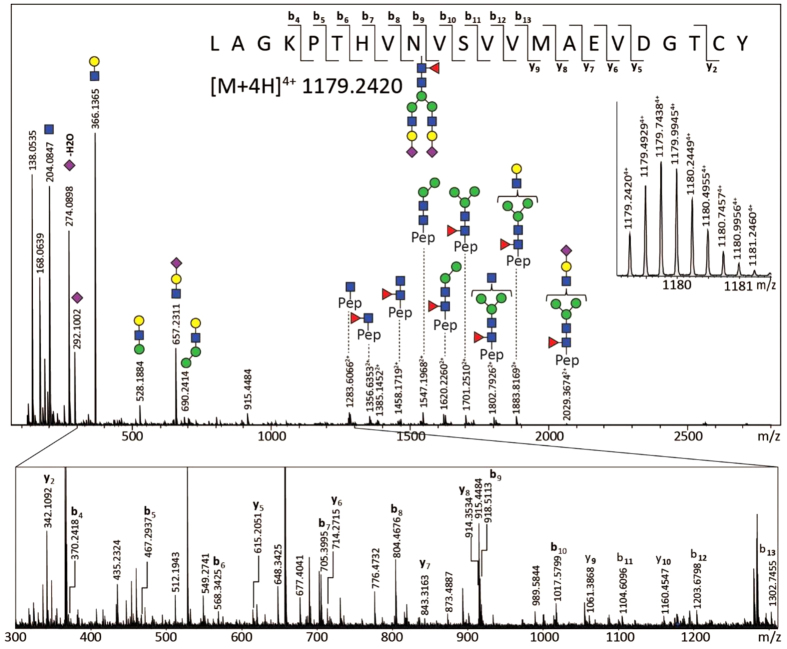
An ESI-QTOF-MS/MS fragmentation spectrum of an Asn340 non-truncated tryptic glycopeptide [M+4H]^4+^ of m/z 1179.2420. The spectrum was acquired in combined lower- and higher-energy CID, exhibiting glycan fragmentation (upper panel) and also fragmentation of the peptide backbone (zoom-in lower panel).

An analyte list containing the observed putative glycopeptides was curated on the basis of overall abundance and QC-values. The identity of some *O*-glycopeptide species was confirmed by ESI-FTICR-MS(/MS), and *N*-glycopeptide confirmation was obtained by C18-nanoLC-quadrupole-TOF-MS/MS. The major glycovariants of each glycopeptide cluster were thus identified (Fig. 3, Supplementary Table S2, and Supplementary Fig. S2). Of note, for the *O*-glycopeptides no site assignment has been performed. The *N*-glycopeptide moieties were confirmed by performing a glycan release on trypsin digested IgA, and analysing these digests on LC-ESI-MS/MS, followed by a proteomics data analysis where deamidation was set as a variable modification (Supplementary Methods, Supplementary Table S1). Finally, *N*-glycan structures were confirmed by analysing ethyl esterified released glycans using MALDI-TOF-MS (Supplementary Methods, Supplementary Fig. S4), and *O*-glycan structures by HPLC^7^. The released *N*-glycan analysis additionally indicated that the sialic acids were almost exclusively α2,6-linked.

The combined MS and MS/MS data for both glycopeptides and glycans furthermore indicated the absence of difucosylated species, which would, if present, in part overlap with sialylated species. Most of the analysed samples showed a partial oxidation of the glycopeptides covering site Asn340. While the assignment of most of the peaks was straight-forward, oxidized, monofucosylated species, which are isomeric with species that instead contain an additional hexose, were slightly more challenging. However, by using the released glycan information in most cases unambiguous assignment was achieved, which was illustrated by highly similar oxidation ratios for all observed oxidized/non-oxidized glycopeptide pairs (data not shown).

Altogether, this resulted in a final list of 53 *O*-glycopeptide conformations, 6 glycoforms for the Asn144 containing *N*-glycopeptide, and 13 glycoforms for the Asn340 containing glycopeptides, of which 3 glycoforms were observed for both the truncated and the non-truncated peptide variant, and 10 only for the truncated peptide (Table 1; Supplementary Table S3). For the *O*-glycopeptide cluster (approximately *m/z* 5350–8000) an average mass measurement error after internal calibration of 1.72 ppm was obtained. The *N*-glycopeptide clusters (approx. *m/z* 3900–5350) similarly showed low- to sub-ppm average mass measurement errors: Asn340, 0.94 ppm; truncated Asn340, 1.28 ppm; Asn144, 1.37 ppm.

### MALDI-FTICR-MS spectrum selection

The glycopeptide clusters were subsequently subjected to a quality check (see Methods section for details). Robustness of the method was then tested using a standard plasma sample along with the clinical samples. The standard samples were included at least in triplicate on each 96-wells plate. For all glycopeptide clusters the inter plate variation of the standard was below 20% (Supplementary Table S4), while the biological variation was on average almost twice as high.

After spectra curation the final dataset contained 329 hinge region *O*-glycosylation profiles, 292 Asn144 *N*-glycosylation profiles, and 329 Asn340 *N*-glycosylation profiles. When two profiles of the same glycopeptide cluster were available for a sample, the highest intensity variant was used for the analysis. Only patients for whom spectra of all time points were available were included in the final statistical analysis. Calculated *O*-glycosylation traits could be analysed pairwise for 27 individuals, traits from Asn144 for 25 individuals, and from Asn340 glycopeptides the traits could be analysed pairwise for 23 (non-truncated peptide) and 25 individuals (truncated peptide).

### Site specific changes in IgA *N*-glycosylation during pregnancy and after delivery

**Figure 4:**
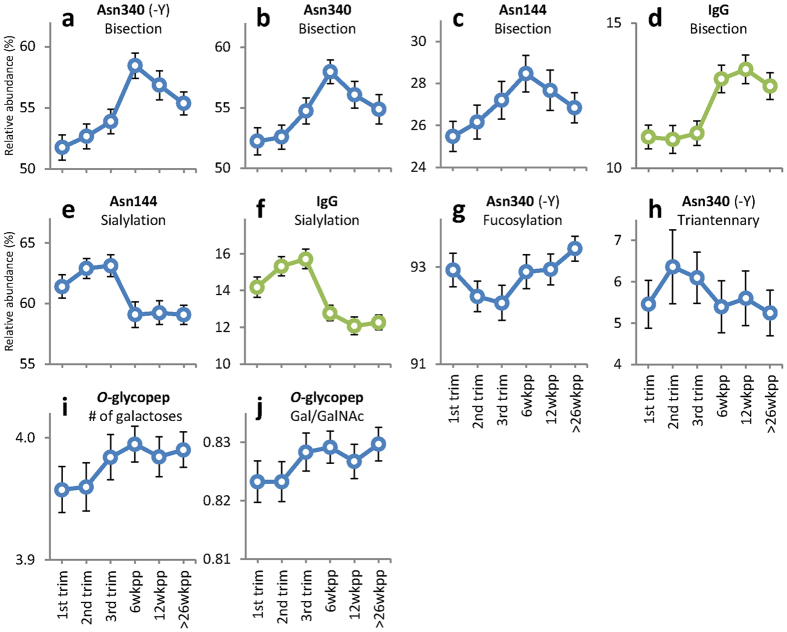
Observed IgA glycosylation changes over time during pregnancy and after delivery for the calculated traits. IgG data is included for comparative reasons (taken from Ref. 38). *N*-glycosylation: levels of bisecting GlcNAc (**a–c**; comparison with IgG in **d**), sialylation (**e**; comparison with IgG in **f**), and fucosylation (**g**) on diantennary complex type glycans, and level of triantennary glycans (**h**). *O*-glycosylation: number of galactoses per peptide (**i**) and the ratio of Galactoses per GalNAc (**j**). Depicted are the mean values per time point, error bars represent standard errors.

The level of bisection of the diantennary glycans on Asn340 increased during pregnancy (truncated peptide: 52% to 54%, p = 0.0001, Fig. 4a; non-truncated: 52% to 55%, p = 0.0072, Fig. 4b). Notably, the increase of bisection observed during pregnancy continued after delivery, and highest bisection levels were observed at 6 weeks postpartum (58% as compared to 55% in the 3^rd^ trimester; p = 0.0002). Similarly, Asn144 bisection showed its maximum at 6 weeks postpartum (28%, Fig. 4c).

Sialylation of Asn144 was higher during pregnancy than after delivery (63% vs. 59%, p = 0.0002; Table 2, Fig. 4e). The decrease occurred rapidly between the last time point of pregnancy and 6 weeks postpartum, after which it remained lowered. In contrast, for Asn340 no changes in the level of sialylation (>89%) were observed.

The tailpiece glycan fucosylation showed a minor, but significant, decrease during pregnancy, and an increase after delivery (p < 0.002, Fig. 4g). This was not reflected on the Asn144 *N*-glycosylation site, where no fucosylated glycans were observed.

The larger variety of *N*-glycans on Asn340 compared to Asn144 was further illustrated by the presence of triantennary glycans. The abundance of these glycans increased during pregnancy (5 to 6%, p = 0.0017, Fig. 4h). After delivery the levels dropped to 5% (p < 0.008).

Both *N*-glycosylation sites of IgA1 showed complete or near complete levels of galactosylation, for which no changes were observed during pregnancy, nor after delivery.

Values for all calculated *N*- and *O*-glycosylation traits at all time points, and the corresponding standard errors, are depicted in Supplementary Table S5.

### Pregnancy associated changes in IgA *O*-glycosylation

The *O*-glycosylation of the IgA1 hinge region remained fairly stable throughout pregnancy and the time after delivery. The numbers of GalNAc residues and sialic acids did not change, and neither did the number of sialic acids per galactose. Very small yet significant increases of the number of galactoses (3.96 to 3.98, p = 0.0095; Table 2, Fig. 4i) and the related ratio of galactoses per GalNAc (0.82 to 0.83, p = 0.0109, Fig. 4j) during pregnancy were observed.

## Discussion

In the current manuscript we describe a method for the simultaneous analysis of IgA1 *N*- and *O*-glycosylation using affinity purification and a simple trypsin digestion, followed by a two-step HILIC enrichment of the glycopeptides, and MALDI-FTICR-MS detection. The high-throughput technique is estimated to allow for the analysis of 384 serum samples within 24 h. Fifty-three O-glycopeptide compositions, 6 Asn144 glycoforms and 13 Asn340 glycoforms can be distinguished with high mass accuracy (average <2 ppm error). The technique was applied on a small pregnancy cohort, showing several changes during pregnancy and/or after delivery.

Only a limited number of groups performed MALDI-MS analysis of IgA glycosylation before^5^,^6^,^11^. These MALDI-TOF-MS experiments were performed in low resolution linear mode, and mass precision was not stated^5^, or large mass deviations were allowed: ±1 Da (140–250 ppm; *m/z* window 3500–8000) for *O*-glycopeptide analysis^6^, and 0.3% (3000 ppm) for sialidase treated *N*-glycopeptides^11^. Of note, Tarelli *et al*. did not analyse tryptic peptides; therefore only the compositions can be compared to our data, and not the observed masses. Nevertheless, a large body of correctly annotated glycopeptide was thus gathered (Table 1; Supplementary Table S3). Generally, we were able to gain more information in the high mass region, whereas previous publications observed more low mass *O*-glycopeptides. We observed five *O*-glycopeptide compositions that have not been published previously. This may be caused by different sample preparation, for example a slight skewing of the spectra to higher masses because of the HILIC-SPE, or due to limited in-source fragmentation in our intermediate pressure FTICR mass spectrometer.

In addition, the use of an ultrahigh resolution MALDI-FTICR-MS circumvented LC steps - generally taking 30 to 120 min per sample, e.g.^6^ - prior to the MS measurement. For the MALDI approach a simple 2-step HILIC was necessary, taking only 60 minutes for 96 samples. The second step of the HILIC was necessary to obtain data concerning the Asn144 containing glycopeptide. However, this manual step can be automated to reduce hands-on time. Moreover, the intermediate pressure in the FTICR prevents the loss of sialic acids observed in MALDI-TOF-MS^32^,^36^. Generally, the measurements and relative quantitation of IgA1 *O*-glycosylation in MALDI mode has been performed on de-sialylated glycopeptides^17^, or on low-level sialylated myeloma species^37^, thus losing the valuable information about the sialic acids. MALDI-MS analysis of the intact *N*-glycopeptides to obtain site-specific information has only been published once^11^. Again, sialidase treated glycopeptides were analysed. The technique described in the current manuscript allows to analyse intact *N*- and *O*-glycopeptides bearing sialic acids in a high-throughput MALDI-MS approach. This allowed for the discovery of eight glycopeptides containing the Asn340 site, generally with higher sialylation compared to those reported in literature (Supplementary Table S3). Among these are some triantennary structures. Furthermore, a few non-fucosylated glycoforms were newly detected. Interestingly, the described technique without sialidase treatment allowed for the specific detection of non-sialylated species on Asn144 as well.

We observed the Asn340 containing glycopeptide with a truncated C-terminus, which is in accordance with previous reports^18^,^30^. In our data the truncated version is more abundant than the non-truncated version, which is reflected in the higher spectral count in our proteomics analysis (42 vs. 16 counts). We assume that this truncation is naturally occurring on IgA from the circulation, and is not induced during storage or sample preparation. However, it is unclear which protease would be responsible for this processing, and whether it is of functional significance.

Both IgA subclasses (i.e. IgA1 and IgA2 isoforms) were captured in the here described procedure, as concluded from bottom-up protein identifications (Supplementary Table S1). However, IgA2 specific glycopeptides were not detected in our MALDI-FTICR-MS spectra. Likely, this is due to the rather low abundance of this subclass (~10%). Moreover, the expected masses of glycopeptides related to the IgA2 Asn205 containing tryptic peptide are outside the detection window.

In the current analysis of pregnancy-associated glycosylation changes on the glycopeptide level, we show changes in *N*-glycosylation and – to a lesser extent – *O*-glycosylation traits for IgA1. The site specific approach showed a decrease in sialylation after delivery (63% to 59%) for Asn144, whereas sialylation on Asn340 did not change. On the other hand, there was a pronounced increase in bisection on Asn340 from 1^st^ trimester up to 6 weeks postpartum (52% to 58%), while the same process was observed on Asn144 to lower extent. Notably, our earlier pilot study by Ruhaak *et al*., which in contrast to the present study was performed merely on IgA-derived *N*-glycans, did not reveal pregnancy-associated glycosylation changes, which may be due to the fact that only samples from 6 individuals were analysed^9^. Whether the changes are biologically important needs to be further investigated.

Surprisingly, IgA bisection shows a different time course than IgG bisection. While IgA bisection increases during pregnancy, and even further after delivery, IgG bisection remains at the same low level during pregnancy and only increases after delivery^38^. After the first time point postpartum IgG bisection remains at the same level while IgA bisection decreases after this time point. The differences between IgA and IgG bisection time courses may have to do with their different biological roles and sites of action, yet this will require further research. In general, the role of bisection in modulating antibody function still needs to be clarified for both IgG and IgA.

For IgG-Fc *N*-glycans increased levels of galactosylation and sialylation during pregnancy compared to the non-pregnant state are well described in both healthy and diseased women^38^,^39^,^40^,^41^,^42^. On IgG these changes are restricted to the Fc portion, whereas the Fab portion, carrying more processed glycans, does not exhibit these pregnancy associated changes in galactosylation and sialylation, although for the latter a trend is visible^38^. Similarly, these changes are not observed for the diantennary glycans on Asn340 of IgA1, which are near completely processed with levels of galactosylation and sialylation of >99% and >89%, respectively.

Sialic acids on *N*-glycans of the standard serum IgA samples analysed in this study were almost exclusively α2,6-linked (>95%), which is in line with previous reports^4^,^10^,^25^. With regard to the level of sialylation of *N*-glycans, Orczyk-Pawilowicz *et al*. reported no significant changes after delivery^25^. However, in the current study we have found that sialylation levels at Asn144 were lower after delivery. These differences could be explained by the chosen time points: 6 weeks postpartum in our study versus 2 weeks after delivery in the earlier report. In addition, in this earlier study the glycosylation of IgA was detected by using lectins that are not site-specific. Differences in sialylation occurring at one site (e.g. 4% change at Asn144) can be obfuscated by the high levels (>89%) of sialylation on Asn340, as well as by the highly sialylated *O*-glycans.

In conclusion, we have developed a high-throughput method based on ultrahigh resolution MALDI-FTICR-MS that enables simultaneous profiling of IgA *N*- and *O*-glycosylation at the glycopeptide level. The ultrahigh resolving power allow for accurate detection of more than 50 *O*-glycopeptides and 6 and 13 glycoforms for the Asn144 and Asn340 *N*-glycopeptides, respectively. The suitability of the method to profile clinical samples was demonstrated by the application on a pregnancy cohort to study potential serum IgA glycosylation changes. In addition, this method can be used in conjunction with remodelling of IgA glycosylation and *in vitro* IgA bioactivity assays to study the functional relevance of IgA glycosylation with respect to antibody dependent cellular cytotoxicity and other effector mechanisms.

## Methods

### Human serum and plasma samples

Twenty-nine healthy Caucasian women who participated in the PARA (Pregnancy-induced Amelioration of Rheumatoid Arthritis) study donated serum at the 1^st^, 2^nd^ and 3^rd^ trimester of pregnancy and at six, twelve and >26 weeks postpartum^40^. The PARA study was in compliance with the Helsinki Declaration and was approved by the Ethics Review Board at the Erasmus University Medical Centre, Rotterdam, The Netherlands, and experiments were performed accordingly. Informed consent was obtained from all subjects.

In addition, EDTA plasma from a healthy donor was used as a technical control throughout the measurements.

### Capture of polyclonal IgA

Human polyclonal IgA was captured from 10 μL human serum or plasma using CaptureSelect IgA beads. Twenty microliter bead slurry was applied to each well of a 96-well Orochem filter plate (10 μm pore size). The beads were pre-washed on a vacuum manifold with three times 200 μL PBS. Subsequently, the samples were diluted in 100 μL PBS on the plate, and incubated on a multiwell plate shaker for 1h. The beads with captured IgA were washed on the vacuum manifold with PBS and MQ water (3 × 200 μL), followed by elution using 100 μL 100 mM FA. The eluates were collected by centrifugation (1 min, 50 × *g*) into V-bottom plates. Finally, the IgA samples were dried by vacuum centrifugation.

### Trypsin digestion

The dried samples were reconstituted in 100 μL 20 mM ammonium bicarbonate buffer (pH 8) and reduced with 2 μL 125 mM DTT for 30 min at 60 °C. Sequentially alkylation with 2 μL 200 mM IAM was performed for 30 min at room temperature, followed by overnight digestion of IgA by TPCK treated trypsin at 37 °C, using 600 ng enzyme per well.

### HILIC enrichment and spotting

Obtained trypsin digests of IgA samples were enriched for glycopeptides by a two-step microtip cotton HILIC SPE, using cotton thread as the solid phase, as described before^38^, with minor modifications in the used solution volumes and composition. Briefly, 15 μL of the digest was transferred to a 96 wells V-bottom plate, and adjusted to 70% ACN by addition of 35 μL ACN. The cotton thread was washed three times with 20 μL MQ, and conditioned three times with 20 μL 70% ACN, using a 12-channel micropipette (2–20 μL). The sample was then loaded on the cotton by pipetting up-and-down 20 times. Subsequently, the cotton was washed 3 times with 20 μL 70% ACN containing 1% TFA and three washes with 20 μL 70% ACN, followed by elution in 5 μL MQ. Twenty-five microliter ACN was then added to the tryptic digest to bring the sample to 80% ACN for a sequential HILIC enrichment with 80% instead of 70% ACN, to capture glycopeptides that were not detectable in the 70% HILIC eluates. The eluates from both HILIC experiments were mixed separately with 15 μL 4-chloro-α-cyanocinnamic acid matrix (Cl-CCA; 0.33 mg/mL in acetone:ethanol 1:2) and 1 μL of each eluate was spotted on a Bruker AnchorChip plate with 800 μm anchors. All glycopeptide samples were spotted in duplicate, resulting in 384 spots per 96 serum samples.

### MALDI- and ESI-FTICR-MS

Both MALDI- and ESI-FTICR-MS experiments were performed on a Bruker 15 Tesla solariX^TM^ FTICR mass spectrometer equipped with a CombiSource (Bruker Daltonics, Bremen, Germany) and controlled by Compass solariXcontrol software. For details on the FTICR-MS settings see Supplementary Methods.

For profiling serum IgA1 *N*- and *O*-glycosylation, MALDI-FTICR-MS experiments were performed. Two duplicate spots were measured for each spotted sample. The FTICR system was externally calibrated using a dextran polysaccharide hydrolysate. The MALDI-FTICR-MS method introduced in this manuscript was applied to 174 serum samples from the cohort, 10 standard plasma samples from a healthy volunteer, and 12 blanks. All samples were spotted in duplicate and the whole workup was performed twice. In total 1536 MALDI spectra were obtained. DataAnalysis Software 4.0 SP4 (Bruker Daltonics) was used for the visualization and conversion of the MALDI spectra into .xy files.

For the characterization of some of the *O*-glycopeptides direct infusion ESI-FTICR-MS experiments were carried out using the settings specified in the Supplementary Methods section. Prior to MS/MS experiments the FTICR system was externally calibrated using a commercially available tune mix (Agilent).

### LC-ESI-MS/MS

Additional structure confirmation of the *N*-glycopeptides was performed via nanoRP-LC-QTOF-MS/MS analysis on a high-resolution MaXis QTOF mass spectrometer (Bruker Daltonics) equipped with a captiveSpray nanoBooster source (Bruker Daltonics) coupled to a Ultimate 3000 nanoUPLC system (Dionex/Thermo Scientific, Breda, The Netherlands). The mass spectrometer and the LC were controlled by Hystar 3.2 (Dionex/Thermo Scientific) and data analysis was performed using DataAnalysis 4.2 (Bruker Daltonics). Experimental details on the LC-ESI-MS/MS method are specified in the Supplementary Methods section. MS/MS was performed using an inclusion list for the selected glycopeptides.

### Data processing

The XY data of the MALDI-FTICR experiments were internally recalibrated with MassyTools v1.6.3.0^43^ using a list of four *N*-glycopeptides (3^rd^ isotopic peak of Asn340 (truncated) bearing H5N4F1S1: *m/z* 4245.8192; Asn340 (truncated) H5N4F1S2: *m/z* 4536.9146; Asn340 (truncated) H5N5F1S2: *m/z* 4739.9940; Asn340 H5N5F1S2: *m/z* 4903.0579; oxidized +15.9949 Da) with and without oxidation, and seven *O*-glycopeptides (H4N4S2: *m/z* 6182.6181; H4N5S2: *m/z* 6385.6974; H4N4S3: *m/z* 6473.7135; H4N5S3: *m/z* 6676.7929; H4N5S4: *m/z* 6967.8883; H5N5S4: *m/z* 7129.9411; H5N5S5: *m/z* 7421.0365). The in-house developed tool sequentially integrated the data for all isotopic peaks that theoretically cover 99% of the analyte intensity, and determined the signal-to-noise ratios. In addition, a quality control (QC) value gave an indication of the quality of each analyte signal by analysing the deviation of the observed isotopic pattern from the theoretical pattern. The QC value calculation was adapted not to include noise in the equation.

### Generation of analyte list

Although most literature depicted the *N*-glycans of IgA1 to be highly galactosylated and sialylated diantennary complex type, some also reported high mannose structures, as well as tri- and tetraantennary glycans. The *O*-glycans were found previously to be of various variants of the core 1 type. Therefore we started our analysis with a non**-curated list of *N*- and *O*-glycopeptides. The list contained 65 potential *N*-glycoforms for each *N*-glycopeptide. Three *N*-glycopeptide sequences were included, namely LSLHRPALEDLLLGSEANLTCTLTGLR for Asn144, and LAGKPTHVNVSVVMAEVDGTCY for Asn340, the latter with and without a truncated tyrosine (Y) at the *C*-terminus. Finally, for the Asn340 glycopeptides oxidation of the methionine (Met345) was included as a potential variable. In addition 182 potential *O*-glycopeptides (ranging from 1 HexNAc up to 6 HexNAc + 6 Hex + 8 SA) were included for extraction, considering a maximum of six occupied sites with core 1 *O*-glycans on the peptide HYTNPSQDVTVPCPVPSTPPTPSPSTPPTPSPSCCHPR. In total the list of potential analytes contained 507 (182+65+65+65+65+65) structures. The theoretical *m/z* values of the truncated Asn340 containing glycopeptide (both oxidized and non-oxidized) bearing a Man5 or H3N4 glycan were outside the measuring range and were therefore excluded.

In order to curate the list of analytes the following steps were taken: analytes showing signal-to-noise greater than six in less than 50% of the spectra were removed from the analysis. In addition, the 25% quartile boundary of the QC value distribution had to be smaller than 0.03 for an analyte to be included in the final extraction. When two analytes were overlapping, the analyte with the lowest QC value was selected. In case of small differences in QC values, the expectancy based on the released *N*-glycan relative abundance was used to select the analyte. Finally, the internally calibrated spectra for the technical controls and for the healthy pregnant women were summed, resulting in two sum spectra for the analysis of the *O*- and the Asn340 *N*-glycopeptide clusters, and two for the Asn144 *N*-glycopeptide cluster. Again, the QC values for the analytes had to be <0.03 for analytes to be included. Finally, the mass accuracy errors of the observed species were inspected to confirm the identity of the extracted species. A median error of less than 5 ppm was set as an inclusion threshold. In the final analyte list overlapping *N*-glycopeptides were curated based on the relative abundances of released glycans, which were obtained as described in the Supplementary Methods.

### Quality threshold for MALDI-FTICR-MS spectra

In order to obtain high quality data, a curation step was performed on the obtained MALDI-FTICR-MS spectra. The total intensity per glycopeptide cluster was calculated based on the final analyte list. In addition, the percentage of glycopeptides with a signal-to-noise ratio (S/N) greater than six was calculated for each of the clusters. The total intensity was then plotted against the percentage with S/N >6 (example in Supplementary Fig. S1). This plot was used as a guide to set cut-off values for both total intensity and the percentage. As a readout the average relative standard deviation per time point was monitored, as well as the percentage of spectra passing the cut-off threshold for which approximately 90% was considered as the lower limit. Finally, from each sample the highest intensity spectrum of the duplicates was selected.

### Calculation of glycosylation traits

*O*-glycosylation features were calculated for each sample: the number of GalNAcs, galactoses, sialic acids, as well as the ratios for sialic acids per galactose and galactose per GalNAc. In addition, we checked the relative abundances of peptides with more GalNAcs than galactoses, indicative for the presence of Tn-antigen, and those with more sialic acids than galactoses. For all the *N*-glycopeptides sialylation and bisection of diantennary glycans was calculated. For the truncated Asn340 containing peptide additionally galactosylation and fucosylation of diantennary glycans, and the abundance of triantennary glycans were calculated. The formulas used to calculate all the derived traits are shown in the Supplementary Methods.

### Statistical analysis

The Wilcoxon signed-rank test was performed for paired samples without assumptions about normality. To correct for the three tests within each calculated glycosylation trait a Bonferroni correction for multiple testing was performed, with p < 0.017 considered as significant.

## Additional Information

**How to cite this article**: Bondt, A. *et al*. Longitudinal monitoring of immunoglobulin A glycosylation during pregnancy by simultaneous MALDI-FTICR-MS analysis of *N*- and *O*-glycopeptides. *Sci. Rep*. **6**, 27955; doi: 10.1038/srep27955 (2016).

## Supplementary Material

Supplementary Information

## Figures and Tables

**Figure 1:**
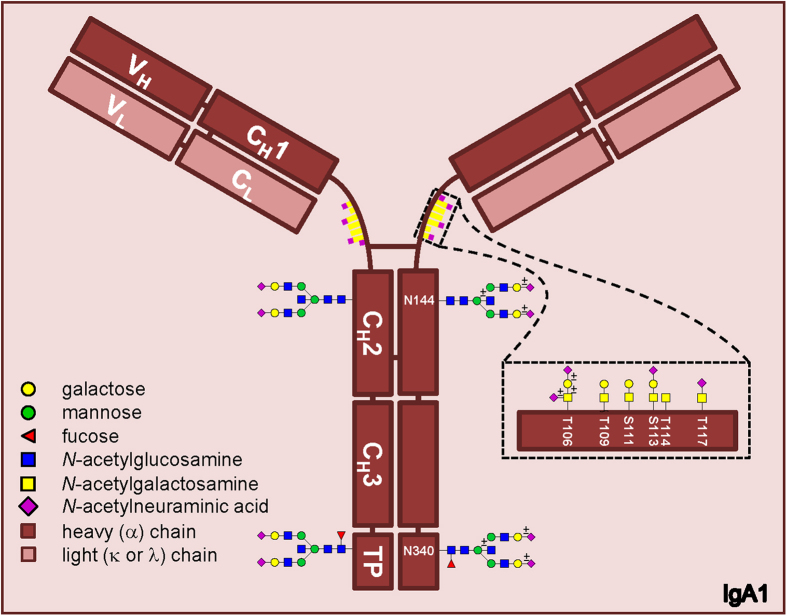
Schematic representation of IgA1 showing two *N*-glycosylation sites on the heavy chain, and hinge-region *O*-glycosylation. The inset shows several possible core 1 (T-antigen) and Tn antigen *O*-glycan structures. The ± symbol indicates that the outer monosaccharide may or may not be present.

**Table 1:** Detected *O*-glycopeptides with the corresponding monoisotopic theoretical mass and median observed ppm error.

*O*- glycopeptide compositions	*m/z*	Error (ppm)	Literature
H2N3S1	5361.3285	2.45	5,17
H3N4	5435.3653	−0.10	6,12,17
H3N3S1	5523.3814	1.30	5,6,12,17
H2N4S1	5564.4079	1.43	5,17
H4N4	5597.4181	1.14	5,12,17
H3N5	5638.4447	2.31	6,12,17
H2N3S2	5652.4240	1.81	5,17
H3N4S1	5726.4607	−1.70	5,12,17
H2N5S1	5767.4873	0.04	5,17
H4N5	5800.4975	1.59	12,17
H3N3S2	5814.4768	2.45	5,12,17
H2N4S2	5855.5033	3.79	5,17
H4N4S1	5888.5136	1.12	5,6,12,17
H3N5S1	5929.5401	−0.65	5,6,17
H3N4S2	6017.5562	−0.91	5,6,12,17
H2N5S2	6058.5827	0.06	5,6,17
H4N5S1	6091.5929	−0.33	6,12
H3N3S3	6105.5722	0.61	6,17
H4N4S2	6179.6090^a^	2.44	5,6,12,17
H3N5S2	6220.6355	1.62	5,6,12,17
H5N5S1	6253.6458	−1.12	12
H4N6S1	6294.6723	−0.68	12
H3N4S3	6308.6516	−1.89	5,17
H4N5S2	6382.6884^a^	−1.75	5,6,12,17
H3N6S2	6423.7149	−1.14	5,6,17
H4N4S3	6470.7044^a^	−0.80	5,6,12,17
H3N5S3	6511.7309	1.33	5,17
H5N5S2	6544.7412	2.17	5,6,12
H4N6S2	6585.7677	3.07	5,6,12,17
H3N4S4	6599.7470	3.71	5,6
H4N5S3	6673.7838^a^	−0.16	5,6,12,17
H3N6S3	6714.8103	−0.09	5,6,17
H5N6S2	6747.8205	−0.69	12
H4N4S4	6761.7998	−1.02	5,6,12,17
H3N5S4	6802.8264	−2.13	5,6
H5N5S3	6835.8366	−2.02	5,6,12,17
H4N6S3	6876.8631	−1.98	5,6,17
H4N5S4	6964.8792^a^	−0.22	5,6,12,17
H5N6S3	7038.9160	0.95	5
H4N4S5	7052.8952	1.55	6,17
H5N5S4	7126.9320^a^	3.41	5,6,12,17
H4N6S4	7167.9586	4.09	5,17
H4N5S5	7255.9746	2.38	5,6,17
H5N6S4	7330.0114	2.73	5
H4N4S6	7343.9906	2.79	
H5N5S5	7418.0274^a^	−1.65	5,6,17
H4N6S5	7459.0540	−3.31	
H6N6S4	7492.0642	−1.06	
H4N5S6	7547.0700	−1.77	
H5N6S5	7621.1068	−1.09	6
H5N5S6	7709.1228	−1.55	6
H6N6S5	7783.1596	−1.40	
H5N5S7	8000.2183	2.00	

Additionally literature references are depicted for previously observed glycoforms. Abbreviations: H = hexose; N = N-acetylhexosamine; S = *N*-acetylneuraminic acid; n.d. = not detected; a = 4th isotopic peak used for calibration.

**Table 2:** Mean and standard error of the calculated glycosylation traits for the time points used in the statistical analysis.

		trim1*		trim3		6wkpp		26wkpp		*n*	trim1 vs trim3	trim3 vs 6wkpp	trim3 vs 26wkpp
		%	SE	%	SE	%	SE	%	SE				
Asn 144	Sialylation	61.39	0.96	63.12	0.90	59.08	1.05	59.04	0.80	25	ns	0.0002	0.0002
	Bisection	25.47	0.72	27.20	0.90	28.47	0.88	26.85	0.72	25	0.0119	ns	ns
Asn 340	Sialylation	95.18	0.17	95.35	0.16	95.40	0.19	95.22	0.26	23	ns	ns	ns
	Bisection	52.24	1.12	54.67	1.07	57.98	0.99	54.88	1.24	23	0.0072	0.0020	ns
Asn340 (truncated)	Galactosylation	99.84	0.01	99.83	0.01	99.82	0.01	99.84	0.01	25	ns	ns	ns
	Sialylation	89.56	0.21	89.12	0.19	89.26	0.23	89.25	0.27	25	ns	ns	ns
	Fucosylation	92.94	0.34	92.26	0.36	92.90	0.35	93.38	0.26	25	0.0007	0.0014	0.0005
	Bisection	51.76	1.04	53.87	1.00	58.45	1.03	55.36	0.94	25	0.0001	0.0000	ns
	Triantennary	5.46	0.26	6.10	0.28	5.40	0.29	5.24	0.25	25	0.0017	0.0009	0.0080
		**#**	**SE**	**#**	**SE**	**#**	**SE**	**#**	**SE**				
*O*-glycosylation	Number of GalNAcs	4.81	0.01	4.81	0.01	4.82	0.01	4.81	0.01	27	ns	ns	ns
	Number of Gal	3.96	0.02	3.98	0.02	3.99	0.01	3.99	0.01	27	0.0095	ns	ns
	Number of SA	3.03	0.03	3.02	0.03	3.04	0.02	3.05	0.03	27	ns	ns	ns
	SA per Gal	0.77	0.01	0.76	0.01	0.76	0.01	0.77	0.01	27	ns	ns	ns
	Gal per GalNAc	0.82	0.004	0.83	0.003	0.83	0.003	0.83	0.003	27	0.0109	ns	ns
		**%**	**SE**	**%**	**SE**	**%**	**SE**	**%**	**SE**				
	% with more GalNAc than Gal	61.36	0.94	60.27	0.86	59.99	0.76	59.81	0.79	27	ns	ns	ns
	% with more SA than Gal	6.49	0.43	6.79	0.36	6.69	0.30	6.59	0.31	27	ns	ns	ns

In addition the number of paired samples for the Wilcoxon signed-rank test are depicted, as well as the resulting p-values. P < 0.0167 was considered as statistically significant after Bonferroni correction.

^*^Abbreviations: trim = trimester; wkpp = weeks postpartum; GalNAc = *N*-acetylgalactosamine; Gal = galactose; SA = *N*-acetylneuraminic acid.
