# Supplementary material for: Longitudinal monitoring of immunoglobulin A glycosylation during pregnancy by simultaneous MALDI-FTICR-MS analysis of N- and O-glycopeptides
Source: Sci Rep. 2016 Jun 15;6:27955. doi: 10.1038/srep27955 (PMC4908400; doi:10.1038/srep27955)
Supplement: Supplementary Information [file srep27955-s1.pdf]

Longitudinal monitoring of immunoglobulin A glycosylation during pregnancy by simultaneous MALDI-FTICR-MS analysis of *N*- and *O*-glycopeptides

Albert Bondt[1,2,*], Simone Nicolardi[2,§], Bas C. Jansen[2,§], Kathrin Stavenhagen[3], Dennis Blank[2], Guinevere S.M. Kammeijer[2], Radoslaw P. Kozak[4], Daryl L. Fernandes[4], Paul J. Hensbergen[2], Johanna M.W. Hazes[1], Yuri E.M. van der Burgt[2], Radboud J.E.M. Dolhain[1], Manfred Wuhrer[2,3,5]

[1] Department of Rheumatology, Erasmus University Medical Center, Rotterdam, The Netherlands
[2] Center for Proteomics and Metabolomics, Leiden University Medical Center, Leiden, The Netherlands
[3] Division of BioAnalytical Chemistry, VU University Amsterdam, The Netherlands
[4] Ludger, Culham Science Centre, Oxfordshire OX14 3EB, UK
[5] Department of Molecular Cell Biology and Immunology, VU University Medical Center, Amsterdam, The Netherlands

[*] Correspondence to: Albert Bondt, PO Box 9600, 2300 RC Leiden; a.bondt@lumc.nl; tel. +31 (0)71 52 68701

Supplementary Material S1
Supplementary Tables S1-S5
Supplementary Figures S1-S4
Supplementary Methods

Supplementary Material S1 – alignment of UniProt protein sequences for the heavy chains of IgA1 (P01876 in blue) and IgA2 (P01877 in green) Potential *N*-linked glycosylation sites are marked in yellow and by underlining (N-X-S/T; X≠P); literature based *O*-linked glycosylation sites are marked in pink and by underlining. Sequence differences on IgA2 are depicted by italic font. Cysteines mentioned on UniProt to be involved in disulphide bonds are depicted in red. In addition, the disulphide links are mentioned below.

```
>sp|P01876|IGHA1_HUMAN Ig alpha-1 chain C region OS=Homo sapiens GN=IGHA1 PE=1 SV=2
>sp|P01877|IGHA2_HUMAN Ig alpha-2 chain C region OS=Homo sapiens GN=IGHA2 PE=1 SV=3

  1 ASPTSPKVFP LSLCSTQPDG NVVIACLVQG FFPQEPLSVT WSESGQGVTA
  1 ASPTSPKVFP LSLDSTPQDG NVVVACLVQG FFPQEPLSVT WSESGQNVTA

 51 RNFPPSQDAS GDLYTTSSQL TLPATQCLAG KSVTCHVKHY TNPSQDVTVP
 51 RNFPPSQDAS GDLYTTSSQL TLPATQCPDG KSVTCHVKHY TNPSQDVTVP

101 CPVPSTPPTP SPSTPPTPSP SCCHPRLSLH RPALEDLLLG SEANLTCTLT
101 CPVPPPPPCC HP                   RLSLH RPALEDLLLG SEANLTCTLT

151 GLRDASGVTF TWTPSSGKSA VQGPPERDLC GCYSVSSVLP GCAEPWNHGK
138 GLRDASGATF TWTPSSGKSA VQGPPERDLC GCYSVSSVLP GCAQPWNHGE

201 TFTCTAAYPE SKTPLTATLS KSGNTFRPEV HLLPPPSEEL ALNELVTLTC
188 TFTCTAAHPE LKTPLTANIT KSGNTFRPEV HLLPPPSEEL ALNELVTLTC

251 LARGFSPKDV LVRWLQGSQE LPREKYLTWA SRQEPSQGTT TFAVTSILRV
238 LARGFSPKDV LVRWLQGSQE LPREKYLTWA SRQEPSQGTT TFAVTSILRV

301 AAEDWKKGDT FSCMVGHEAL PLAFTQKTID RLAGKPTHVN VSVVMAEVDG TCY
288 AAEDWKKGDT FSCMVGHEAL PLAFTQKTID RMAGKPTHVN VSVVMAEVDG TCY
```

```
IgA1 Cys14             Interchain met light chain
     Cys26-85          Intrachain
     Cys77-101         Intrachain
     Cys122            Interchain with heavy chain
     Cys123-180 (182)  Intrachain
     Cys147-204        Intrachain
     Cys182 (180)      Interchain with heavy chain
     Cys192            Interchain with heavy chain of other subunit
     Cys250-313        Intrachain
     Cys352            Interchain with J-chain
IgA2 Cys26-85          Intrachain
     Cys101            Interchain with light chain
     Cys109            Interchain with heavy chain
     Cys110-167        Intrachain
     Cys134-191        Intrachain
     Cys169            Interchain with heavy chain
     Cys179            Interchain with heavy chain of other subunit
     Cys237-300        Intrachain
     Cys339            Interchain with J-chain
```

Supplementary Table S1 Mascot search results obtained from LC-MS/MS for an IgA tryptic digest (**A**) and a deglycosylated tryptic digest (**B**).

A

Protein ID	Assigned peptide sequence	Precursor charge	Precursor m/z	modification	Max. score	All possible other proteins for a peptide
Ig alpha-1 chain C region (P01876; prot.score:911; #pep.:13; seq.cov.:45%)	NFPPSQDASGDLYTTSSQLTLPATQC LAGK	4+	793.267	Deamidated (2×)	41	U[a]
	DASGVTFTWTPSSGK	2+	771.011		67	U
	SAVQGPPER	2+	470.793		53	P01877
	DLCGCYSVSSVLPGCAEPWNHGK	3+	865.486		91	U
	TFTCTAAYPESK	2+	688.395		45	U
	TPLTATLSK	2+	466.310		47	U
	WLQGSQELPR	2+	607.430		67	P01877
	YLTWASR	2+	488.816		33	P01877
	QEPSQGTTTFAVTSILR	2+	918.584		89	P01877
	VAAEDWK	2+	409.790		44	P01877
	GDTFSCMVGHEALPLAFTQK	3+	737.480		36	P01877
Ig alpha-2 chain C region (P01877; prot.score:420; #pep.:8; seq.cov.:32%)	HYTNPSQDVTVPCPVPPPPPCCHPR	4+	728.141		37	U
	DASGATFTWTPSSGK	2+	756.963		44	U
	SAVQGPPER	2+	470.793		53	P01876
	WLQGSQELPR	2+	607.430		67	P01876
	YLTWASR	2+	488.816		33	P01876
	QEPSQGTTTFAVTSILR	2+	918.571		87	P01876
	VAAEDWK	2+	409.790		44	P01876
	GDTFSCMVGHEALPLAFTQK	3+	737.480		36	P01876

[a] U indicates that the peptide is unique for this protein.

Serum albumin (P02768; prot.score:808; #pep.:21; seq.cov.:40%)	DLGEENFK	2+	476.295		31	U
	ALVLIAFAQYLQQCPFEDHVK	3+	831.574	Deamidated	65	U
	LVNEVTEFAK	2+	575.354		48	U
	TCVADESAENCDK	2+	749.856		97	U
	SLHTLFGDK	2+	509.322		36	U
	ETYGEMADCCAK	3+	479.238		42	U
	DDNPNLPR	2+	470.779		38	U
	YLYEIAR	2+	464.340		52	U
	AAFTECCQAADK	2+	686.374		52	U
	AEFAEVSK	2+	440.778		40	U
	VHTECCHGDLLECADDR	4+	552.615		33	U
	YICENQDSISSK	2+	722.389		61	U
	CCAAADPHECYAK	2+	776.881		56	U
	VFDEFKPLVEEPQNLIK	3+	682.504		54	U
	QNCELFEQLGEYK	2+	829.455		59	U
	FQNALLVR	2+	480.847		53	U
	KVPQVSTPTLVEVSR	3+	547.385		56	U
	CCTESLVNR	2+	569.810		34	U
	RPCFSALEVDETYVPK	3+	637.773		44	U
	QTALVELVK	2+	500.860		37	U
	AVMDDFAAFVEK	2+	671.964		61	U
	LVAASQAALGL	2+	507.389		77	U
Ig kappa chain C region (A0A087X130; prot.score:763; #pep.:6; seq.cov.:40%)	LLIYGASTR	2+	497.352		46	U
	TVAAPSVFIFPPSDEQLK	2+	973.618		54	U
	SGTASVVCLLNNFYPR	2+	900.022		50	U
	VDNALQSGNSQESVTEQDSK	2+	1068.567		78	U
	DSTYSLSSTLTLSK	2+	752.033		64	U
	VYACEVTHQGLSSPVTK	3+	626.060		57	U
Ig lambda-2 chain C regions (A0A075B6K9; prot.score:670; #pep.:5; seq.cov.:74%)	AAPSVTLFPPSSEELQANK	2+	994.045		45	U

	ATLVCLISDFYPGAVTVAWK	2+	1106.697		79	U
	AGVETTTPSK	2+	495.808		62	U
	YAASSYLSLTPEQWK	2+	872.561		71	U
	SYSCQVTHEGSTVEK	2+	856.468		62	U
Protein IGHV3-23 (A0A087WSX3; prot.score:166; #pep.:2; seq.cov.:18%)	NTLYLQMNSLR	2+	676.930		72	P01768; A0A075B7B8
	AEDTAVYYCAK	2+	645.876		71	U
Ig heavy chain V-III region CAM (P01768; prot.score:141; #pep.:2; seq.cov.:18%)	NTLYLQMNSLR	2+	676.930		72	A0A087WSX3; A0A075B7B8
	AENTAVYYCAR	2+	659.883	Deamidated	57	U
Protein IGHV3OR16-12 (A0A075B7B8; prot.score:140; #pep.:2; seq.cov.:18%)	NTLYLQMNSLR	2+	676.930		72	A0A087WSX3; P01768
	VEDTAVYYCAR	2+	673.874		62	U
Protein IGHV3OR16-9 (S4R460; prot.score:115; #pep.:2; seq.cov.:31%)	EVQLVESGGGLVQPGGSLR	2+	941.588		73	U
	NSLYLQMNSLR	2+	669.863		58	A0A087X2C0; A0A087WV47
Ig mu chain C region (A0A087X2C0; prot.score:112; #pep.:10; seq.cov.:23%)	NSLYLQMNSLR	2+	669.863		58	S4R460; A0A087WV47
	AEDTAVYYCAR	2+	660.395		56	A0A087WV47
	YAATSQVLLPSK	2+	639.450		42	U
	VSVFVPPR	2+	450.880		41	U
	LICQATGFSPR	2+	625.389		34	U
	QVGSGVTTDQVQAEAK	2+	809.556		35	U
	FTCTVTHTDLPSPLK	3+	573.017		34	U
	GVALHRPDVYLLPPAR	3+	592.442		37	U
	ESATITCLVTGFSPADVFVQWMQR	3+	915.822	Deamidated	36	U
	YVTSAPMPEPQAPGR	2+	800.934		41	U
Ig gamma-1 chain C region (A0A087WV47; prot.score:91; #pep.:5; seq.cov.:11%)	NSLYLQMNSLR	2+	669.863		58	S4R460; A0A087X2C0
	AEDTAVYYCAR	2+	660.395		56	A0A087X2C0
	DTLMISR	2+	418.299		40	U
	FNWYVDGVEVHNAK	3+	560.075		46	U

	NQVSLTCLVK	2+	581.424		40	U
Transthyretin (A0A087WT59; prot.score:130; #pep.:3; seq.cov.:25%)	GSPAINVAVHVFR	2+	684.019		70	U
	AADDTWEPFASGK	2+	697.908		61	U
	TSESGELHGLTTEEEFVEGIYK	3+	819.144		42	U
Ig kappa chain V-III region SIE (P01620; prot.score:128; #pep.:2; seq.cov.:22%)	LLIYGASSR	2+	490.350		55	U
	FSGSGSGTDFTLTISR	2+	817.019		74	U
Alpha-2-macroglobulin (P01620; prot.score:56; #pep.:4; seq.cov.:3%)	NEDSLVFVQTDK	2+	697.941		55	U
	YDVENCLANK	2+	613.895		33	U
	SSGSLLNNAIK	2+	552.416		32	U
	TAQEGDHGSHVYTK	3+	510.669		32	U

B

Protein ID	Assigned peptide sequence	Precursor charge	Precursor m/z	modification	Max. score	All possible proteins for a peptide
Ig alpha-1 chain C region trunc. Y (P01876b; prot.score:2032; #pep.:15; seq.cov.:69%)	NFPPSQDASGDLYTTSSQLTLPATQC LAGK	3+	1057.298		33	P01876
	SVTCHVK	2+	415.808		37	P01876; P01877
	LSLHRPALEDLLLGSEANLTCTLTGLR	3+	988.942	Deamidated	110	P01876; P01877
	DASGVTFTWTPSSGK	2+	770.942		75	P01876
	SAVQGPPER	2+	470.793		49	P01876; P01877
	DLCGCYSVSSVLPGCAEPWNHGK	3+	865.246		60	P01876
	TFTCTAAYPESK	2+	688.346		47	P01876
	TPLTATLSK	2+	466.392		44	P01876
	SGNTFRPEVHLLPPPSEELALNELVTL TCLAR	4+	894.591	Deamidated	30	P01876; P01877

	WLQGSQELPR	2+	607.407		70	P01876; P01877
	YLTWASR	2+	488.822		44	P01876; P01877
	QEPSQGTTTFAVTSILR	2+	918.619		86	P01876; P01877
	VAAEDWK	2+	409.799		44	P01876; P01877
	GDTFSCMVGHEALPLAFTQK	2+	1105.138		54	P01876; P01877
	LAGKPTHVNVSVVMAEVDGTC	3+	729.426	Deamidated	81	U
Ig alpha-1 chain C region (P01876; prot.score:1548; #pep.:15; seq.cov.:69%)	NFPPSQDASGDLYTTSSQLTLPATQCLAGK	3+	1057.298		33	P01876b
	SVTCHVK	2+	415.808		37	P01876b; P01877
	LSLHRPALEDLLLGSEANLTCTLTGLR	3+	988.942	Deamidated	110	P01876b; P01877
	DASGVTFTWTPSSGK	2+	770.942		75	P01876b
	SAVQGPPER	2+	470.793		49	P01876b; P01877
	DLCGCYSVSSVLPGCAEPWNHGK	3+	865.246		60	P01876b
	TFTCTAAYPESK	2+	688.346		47	P01876b
	TPLTATLSK	2+	466.392		44	P01876b
	SGNTFRPEVHLLPPPSEELALNELVTLTCLAR	4+	894.591	Deamidated	30	P01876b; P01877
	WLQGSQELPR	2+	607.407		70	P01876b; P01877
	YLTWASR	2+	488.822		44	P01876b; P01877
	QEPSQGTTTFAVTSILR	2+	918.619		86	P01876b; P01877
	VAAEDWK	2+	409.799		44	P01876b; P01877
	GDTFSCMVGHEALPLAFTQK	2+	1105.138		54	P01876b; P01877
	LAGKPTHVNVSVVMAEVDGTCY	3+	783.484	Deamidated	87	U
Ig alpha-2 chain C region (P01877; prot.score:623; #pep.:11; seq.cov.:47%)	SVTCHVK	2+	415.808		37	P01876;P01876b
	LSLHRPALEDLLLGSEANLTCTLTGLR	3+	988.942	Deamidated	110	P01876;P01876b
	DASGATFTWTPSSGK	2+	756.998		40	U
	SAVQGPPER	2+	470.793		49	P01876;P01876b
	TPLTANITK	2+	480.333	Deamidated	46	U
	SGNTFRPEVHLLPPPSEELALNELVTLTCLAR	4+	894.591	Deamidated	30	P01876;P01876b
	WLQGSQELPR	2+	607.407		70	P01876;P01876b

	YLTWASR	2+	488.822		44	P01876;P01876b
	QEPSQGTTTFAVTSILR	2+	918.619		86	P01876;P01876b
	VAAEDWK	2+	409.799		44	P01876;P01876b
	GDTFSCMVGHEALPLAFTQK	2+	1105.138		54	P01876;P01876b
Serum albumin (P02768; prot.score:798; #pep.:23; seq.cov.:49%)	LVNEVTEFAK	2+	575.404		48	U
	TCVADESAENCDK	2+	749.854		111	U
	SLHTLFGDK	2+	509.356		32	U
	ETYGEMADCCAK	2+	717.879		42	U
	LVRPEVDVMCTAFHDNEETFLK	3+	884.159		46	U
	YLYEIAR	2+	464.311		46	U
	AAFTECCQAADK	2+	686.396		51	U
	AEFAEVSK	2+	440.767		38	U
	VHTECCHGDLLECADDR	4+	552.557		33	U
	YICENQDSISSK	2+	722.405		78	U
	SHCIAEVE<u>N</u>DEMPADLPSLAADFVESK	4+	744.947	Deamidated	31	U
	DVFLGMFLYEYAR	2+	812.536		47	U
	TYETTLEK	2+	492.791		35	U
	CCAAADPHECYAK	2+	776.878		45	U
	VFDEFKPLVEEPQNLIK	2+	1023.108		44	U
	QNCELFEQLGEYK	2+	829.994		66	U
	FQNALLVR	2+	480.796		56	U
	MPCAEDYLSVVLNQLCVLHEK	3+	840.1960		68	U
	CCTESLVNR	2+	569.819		40	U
	RPCFSALEVDETYVPK	3+	637.765		53	U
	QTALVELVK	2+	500.924		47	U
	AVMDDFAAFVEK	2+	671.965		74	U
	LVAASQAALGL	2+	507.390		49	U
Ig kappa chain C region (A0A087X130; prot.score:633; #pep.:6; seq.cov.:40%)	LLIYGASTR	2+	497.377		58	U
	TVAAPSVFIFPPSDEQLK	2+	973.635		50	U

	SGTASVVCLLNNFYPR	2+	899.592		57	U
	VDNALQSGNSQESVTEQDSK	2+	1069.035		99	U
	DSTYSLSSTLTLSK	2+	751.948		64	U
	VYACEVTHQGLSSPVTK	2+	939.067		56	U
Ig lambda-2 chain C regions (A0A075B6K9; prot.score:440; #pep.:5; seq.cov.:63%)	AAPSVTLFPPSSEELQANK	2+	993.587		50	U
	AGVETTTPSK	2+	495.862		62	U
	YAASSYLSLTPEQWK	2+	872.548		57	U
	SYSCQVTHEGSTVEK	2+	856.471		69	U
	TVAPTECS	2+	432.760		35	U
Ig mu chain C region (A0A087X2C0; prot.score:178; #pep.:6; seq.cov.:11%)	AEDTAVYYCAR	2+	659.877		71	A0A087WSX4; A0A087WV47
	YAATSQVLLPSK	2+	639.422		44	U
	VSVFVPPR	2+	450.861		41	U
	QVGSGVTTDQVQAEAK	2+	809.466		73	U
	ESGPTTYK	2+	441.819		31	U
	YVTSAPMPEPQAPGR	2+	800.911		36	U
Protein IGHV3-53 (A0A087WSX4; prot.score:104; #pep.:2; seq.cov.:18%)	NTLYLQMNSLR	2+	677.012		54	U
	AEDTAVYYCAR	2+	659.877		71	A0A087X2C0; A0A087WV47
Ig gamma-1 chain C region (A0A087WV47; prot.score:96; #pep.:5; seq.cov.:13%)	AEDTAVYYCAR	2+	659.877		71	A0A087X2C0; A0A087WV47
	DTLMISR	2+	418.280		37	
	TPEVTCVVVDVSHEDPEVK	3+	713.777		34	U
	FNWYVDGVEVHNAK	3+	560.040		48	U
	NQVSLTCLVK	2+	581.422		41	A0A075B6N8
Ig gamma-3 chain C region (A0A075B6N8; prot.score:51; #pep.:3; seq.cov.:7%)	SCDTPPPCPR	2+	593.816		43	U
	DTLMISR	2+	418.280		37	
	NQVSLTCLVK	2+	581.422		41	A0A087WV47
Ig kappa chain V-I region DEE (P01597; prot.score:120; #pep.:2; seq.cov.:16%)	NIQMTQSPSSLSASVGDR	3+	627.073		50	U

	DIQMTQSPSSLSASVGDR	2+	940.011		89	U
Protein AMBP (P02760; prot.score:93; #pep.:2; seq.cov.:5%)	GVCEETSGAYEK	2+	665.411		67	U
	ETLLQDFR	2+	511.360		52	U
Complement C3 (P01024; prot.score:85; #pep.:3; seq.cov.:3%)	VPVAVQGEDTVQSLTQGDGVAK	3+	733.528		50	U
	ILLQGTPVAQMTEDAVDAER	3+	719.835		48	U
	GYTQQLAFR	2+	542.379		35	U
Alpha-1-antitrypsin (P01009; prot.score:62; #pep.:4; seq.cov.:10%)	LQHLENELTHDIITK	3+	602.085		53	U
	LSITGTYDLK	2+	555.895		33	U
	SVLGQLGITK	2+	508.383		41	U
	AVLTIDEK	2+	444.836		31	U
Ig heavy chain V-III region TUR (P01779; prot.score:62; #pep.:2; seq.cov.:26%)	EVQLLESGGGLVQPGGSLR	3+	632.795		43	U
	LSCAASGFTFSR	2+	652.428		50	U
Protein IGHV4-34 (fragment) (A0A0A0MS12; prot.score:51; #pep.:2; seq.cov.:20%)	VTISVDTSK	2+	475.309		34	U
	LSSVTAADTAVYYCAR	2+	874.544		48	U

Supplementary Table S2: Information gathered from RP-LC-ESI-QTOF-MS/MS analysis of tryptic IgA *N* -glycopeptides (**A**) and ESI-FTICR-MS/MS analysis of the tryptic *O* -glycopeptide bearing H4N4S2 (**B**). Parent masses for the fragmentations are indicated bold; the ions for **B** are derived from two overlapping fragmentation spectra. For the *O* -glycopeptide only compositional confirmation is obtained; glycosylation sites are obtained from literature. Abbreviations: N , *N* -acetylhexosamine; H, hexose; F, fucose; S, N-acetylneuraminic acid; Pep, peptide.

	Peptide sequence	Glycosylation site	Calculated peptide mass [M]	Glycan composition	Observed m/z	Mass difference [ppm]	Observed m/z for pep+GlcNAc $[M+2H]^{2+}$	Mass difference [ppm]	Observed diagnostic ions
**A**	$_{332}$LAGKPTHVNVSVVM(ox)AEVDGTCY$_{353}$	Asn340	2362.1298	H5N4F1S2	$[1571.9857]^{3+}$<br>**$[1179.2420]^{4+}$**<br>$[943.5947]^{5+}$<br>$[786.4967]^{6+}$	5.2<br>4.5<br>4.9<br>5	1283.6069	3.9	b4 370.2418; b5 467.2937; b6 568.3425; b7 705.3995; b8 804.4676; b9 918.5113; b10 1017.5799; b11 1104.6096; b12 1203.6798; b13 1302.7455; y2 342.1092; y5 615.2051; y6 714.2715; y7 843.3163; y8 914.3534; y9 1061.3868; y10 1160.4547; N 204.0847; S-H2O 274.0898; S 292.1002; H1N1 366.1365; H2N1 528.1884; H1N1S1 657.2311; H3N1 690.2414;Pep+N1^{2+} 1283.6066; Pep+N1F1^{2+} 1356.6353; Pep+N2^{2+} 1385.1452; Pep+N2F1^{2+} 1458.1719; Pep+H1N2^{2+} 1466.1725; Pep+H1N2F1^{2+} 1539.1982; Pep+H2N2^{2+} 1547.1968; Pep+H2N2F1^{2+} 1620.2260; Pep+H3N2F1^{2+} 1701.2510; Pep+H2N3F1^{2+} 1721.7628; Pep+H3N3^{2+} 1729.7623; Pep+H3N3F1^{2+} 1802.7926; Pep+H4N3F1^{2+} 1883.8169; Pep+H4N3F1S1^{2+} 2029.3674
				H5N5F1S2	**$[1230.0120]^{4+}$**<br>$[984.2106]^{5+}$<br>$[820.3436]^{6+}$	4.2<br>4.6<br>4.4	1283.6066	4.1	b4 370.2406; b7 705.3997; b8 804.4689; b9 918.5095; b10 1017.5775; b11 1104.6094; b12 1203.6778; b13 1302.7454; y2 342.1091; y5 615.2058; y6 714.2765; N 204.0847; S-H2O 274.0898; S 292.1001; H1N1 366.1364; H2N1 528.1891; H1N1S1 657.2307; H3N1 690.2400; H2N1S1 819.2846; Pep+N1^{2+} 1283.6069; Pep+N1F1^{2+} 1356.6356; Pep+N2^{2+} 1385.1454; Pep+N2F1^{2+} 1458.1720; Pep+H1N2^{2+} 1466.1691; Pep+H1N2F1^{2+} 1539.1947; Pep+H2N2^{2+} 1547.1962; Pep+H1N3^{2+} 1567.7098; Pep+H2N2F1^{2+} 1620.2220; Pep+H1N3F1^{2+} 1640.7399; Pep+H2N3^{2+} 1648.7346; Pep+H2N3F1^{2+} 1721.7661; Pep+H3N3^{2+} 1729.7618; Pep+H3N3F1^{2+} 1802.7943; Pep+H3N4^{2+} 1831.3093; Pep+H4N3F1^{2+} 1883.8107; Pep+H3N4F1^{2+} 1904.3261; Pep+H4N4^{2+} 1912.3384; Pep+H4N4F1^{2+} 1985.3575
	$_{332}$LAGKPTHVNVSVVMAEVDGTC$_{352}$	Asn340	2183.0715	H5N4F1S2	$[1512.3168]^{3+}$<br>**$[1134.4899]^{4+}$**<br>$[907.7938]^{5+}$	6<br>6.3<br>6.8	1194.0899	6	b4 370.2476, b5 467.3015, b6 568.3499; b7 705.4082; b8 804.4787; b9 918.5197; b10 1017.5927; b11 1104.6243; b12 1203.6915; b13 1302.7589; y21 2184.0925; N 204.0886; S-H2O 274.0946; S 292.1052; H1N1 366.1428; H2N 528.1961; H1N1S1 657.2399; H3N 690.2509; H2N1S1 819.2920; Pep+N1^{2+} 1194.0899; Pep+N1F1^{2+} 1267.1202; Pep+N2^{2+} 1295.6288; Pep+H1N2^{2+} 1376.6575; Pep+N2F1^{2+} 1368.6594; Pep+H1N2F1^{2+} 1449.6880; Pep+H2N2^{2+} 1457.6858; Pep+H2N2F1^{2+} 1530.7136; Pep+H3N2^{2+} 1538.7104; Pep+H2N3^{2+} 1559.2318; Pep+H3N2F1^{2+} 1611.7409; Pep+H3N3^{2+} 1640.2526; Pep+H3N3F1^{2+} 1713.2818; Pep+H4N3^{2+} 1721.2785; Pep+H4N3F1^{2+} 1794.3054; Pep+N1 2387.1724; Pep+N1F1 2533.2282
				H5N5F1S2	**$[1185.2604]^{4+}$**<br>$[948.4094]^{5+}$	6.6<br>6.2	1194.0897	5.8	b4 370.2472; b5 467.3027; b6 568.3476; b7 705.4086; b8 804.4779; b9 918.5207; b10 1017.5870; b11 1104.6206; b12 1203.6899; b13 1302.7668; y2 280.0988; y4 452.1473; y5 551.2164; y6 680.2610; y7 751.3012; y8 882.3352; y9 981.4052; N 204.0886; S-H2O 274.0947; S 292.1048; H1N1 366.1426; H2N1 528.1967; H1N1S1 657.2397; H3N1 690.2517; H2N1S1 819.2943; Pep+N12+ 1194.0897; Pep+N1F1^{2+} 1267.1184; Pep+N2^{2+} 1295.6314; Pep+H1N2^{2+} 1376.6598; Pep+N2F1^{2+} 1368.6632; Pep+H1N2F1^{2+} 1449.6906; Pep+H2N2^{2+} 1457.6840; Pep+H1N3^{2+} 1478.1969; Pep+H2N2F1^{2+} 1530.7114; Pep+H3N2^{2+} 1538.7056; Pep+H1N3F1^{2+} 1551.2220; Pep+H2N3^{2+} 1559.2250; Pep+H3N2F1^{2+} 1611.7384; Pep+H3N3^{2+} 1640.2544; Pep+H3N3F1^{2+} 1713.2767; Pep+H4N3^{2+} 1721.2830; Pep+H4N3F1^{2+} 1794.3008; Pep+N1 2387.1760; Pep+N1F1 2533.2272
	$_{332}$LAGKPTHVNVSVVM(ox)AEVDGTC$_{352}$	Asn340	2199.0664	H5N4F1S2	$[1517.6478]^{3+}$<br>**$[1138.4891]^{4+}$**<br>$[910.9927]^{5+}$<br>$[759.3281]^{6+}$	5.5<br>6.7<br>6.7<br>6.2	1202.0883	6.8	b4 370.2476; b5 467.3013; b6 568.3496; b7 705.4087; b8 804.4785; b9 918.5209; b10 1017.5905; b11 1104.6243; b12 1203.6910; b13 1302.7608; b14 1449.7906; y2 280.0984; y3 337.1203; y4 452.1477; y5 551.2172; y6 680.2606; y7 751.2970; y8 898.3343; y9 997.4039; N 204.0888; S-H2O 274.0948; S 292.1052; H1N1 366.1428; H2N1 528.1959; H1N1S1 657.2396; H3N1 690.2499; H2N1S1 819.2943; Pep+N1^{2+} 1202.0883; Pep+N1F1^{2+} 1275.1179; Pep+N2^{2+} 1303.6285; Pep+N2F1^{2+} 1376.6566; Pep+H1N2^{2+} 1384.6553; Pep+H1N2F1^{2+} 1457.6820; Pep+H2N2^{2+} 1465.6820; Pep+H2N2F1^{2+} 1538.7112; Pep+H3N2^{2+} 1546.7095; Pep+H3N2F1^{2+} 1619.7389; Pep+H3N3^{2+} 1648.2491; Pep+H3N3F1^{2+} 1721.2786; Pep+H4N3F1^{2+} 1802.3061; Pep+N1 2403.1650; Pep+N1F1 2549.2251
**B**	$_{89}$HYTNPSQDVTVPCPVPSTPPTPSPSTPPTPSPSCCHPR$_{126}$	Thr106, Thr109, Ser111, Ser113, Thr114, Thr117	4135.8821	H4N4S2	**$[883.6648]^{7+}$**<br>$[1030.7752]^{6+}$<br>**$[1236.7270]^{5+}$**	0.1<br>0.9<br>-0.6	-	-	b9^{2+} 521.7331; b15^{3+} 565.9314; b10^{2+} 572.2570; b11+H2O^{2+} 612.7859; b11^{2+} 621.7911; b11^{1+} 1242.5753; N 204.0867; S+H2O 274.0921; S 292.1027; H1N1 366.1395; H1S1 454.1555; H2N1 528.1923; H1N1S1 657.2349; H2N1S1 819.2878; Pep+H4N4S1^{7+} 842.0795; Pep+H2N3^{6+} 845.8790; Pep+H3N3^{6+} 872.8876; Pep+H2N3S1^{6+} 894.3940; Pep+H3N4^{6+} 906.7340; Pep+H3N3S1^{6+} 921.4026; Pep+H1N2^{5+} 941.8263; Pep+H3N4S1^{6+} 955.2502; Pep+H3N3S2^{6+} 969.9182; Pep+H4N4S1^{6+} 982.4251; Pep+H1N2S1^{5+} 1000.0439; Pep+H2N3^{5+} 1014.8526; Pep+H2N2S1^{5+} 1032.4558; Pep+H3N3^{5+} 1047.2635; Pep+H2N3S1^{5+} 1073.0722; Pep+H3N4^{5+} 1088.0797; Pep+H3N3S1^{5+} 1105.4811; Pep+H3N4S1^{5+} 1146.0985; y27+H4N4S1^{4+} 1162.4935; Pep+H4N4S1^{5+} 1178.5083; Pep+H3N4S2^{5+} 1204.3177; Pep+H2N2^{4+} 1217.5453; Pep+H2N3^{4+} 1268.3147; y27+H2N3^{3+} 1276.8749; Pep+H3N3^{4+} 1308.8292; Pep+H2N3S1^{4+} 1341.0885; Pep+H3N4^{4+} 1359.6004; Pep+H3N3S1^{4+} 1381.6018; Pep+H4N4^{4+} 1400.1111; Pep+H4N3S1^{4+} 1422.1159; Pep+H3N4S1^{4+} 1432.3706; Pep+H3N3S2^{4+} 1454.3757; Pep+H4N4S1^{4+} 1472.8845; y27+H3N4S1^{3+} 1495.6380; y27+H4N4S1^{3+} 1549.6546; y27+H4N4S2^{3+} 1646.6873;

Supplementary Table S3 Detected *N*-glycopeptides with the corresponding monoisotopic theoretical mass and median observed ppm error. Additionally literature references are shown if applicable. Abbreviations: H = hexose; N = *N*-acetylhexosamine; F = fucose; S = *N*-acetylneuraminic acid; n.d. = not detected.

	N-glycan compositions	*m/z*	*Error (ppm)*	Literature
Asn144	H5N4	4586.1793	-1.54	(18)
	H5N4S1	4877.2747	-0.09	(18)
	H5N4S2	5168.3702	1.45	(18)
	H5N5	4789.2587	0.69	(18)
	H5N5S1	5080.3541	-1.50	(18)
	H5N5S2	5371.4495	0.64	
Asn340 (non-trunc.)	H5N4F1S1[a]	4422.8719	0.19	(18)
	H5N4F1S2[a]	4713.9673	-1.59	(18)
	H5N5F1S2[a,b]	4917.0467	-0.62	(18)
Asn340 (trunc.)	H4N4S1	3935.7024	-1.84	
	H5N4S1	4097.7552	-0.13	(18)
	H5N4S1[a]	4113.7501	0.17	
	H5N4S2[a]	4404.8456	-0.90	
	H5N4F1[a]	3968.7126	-1.97	(18)
	H5N4F1S1[b]	4243.8131	0.16	(18)
	H5N4F1S1[a,b]	4259.8080	1.40	
	H5N4F1S2[b]	4534.9086	-0.70	(18)
	H5N4F1S2[a,b]	4550.9035	-0.55	
	H5N5S1	4300.8346	1.01	
	H5N5S1[a]	4316.8295	-1.08	
	H5N5S2	4591.9300	0.64	
	H5N5F1[a]	4171.7920	-1.68	(12,18)[c]
	H5N5F1S1	4446.8925	2.14	(18)[c]
	H5N5F1S1[a]	4462.8874	1.84	
	H5N5F1S2[b]	4737.9879	-0.72	(18)
	H5N5F1S2[a,b]	4753.9828	0.43	
	H6N5F1S1[a]	4624.9402	3.65	
	H6N5F1S3[a]	5207.1311	1.10	(18)
	H6N6[a]	4390.8663	-0.11	

[a] Oxidized peptide
[b] 3rd isotopic peak used for calibration
[c] Only detected non-truncated

Supplementary Table S4 Inter- and intraplate variation observed within a standard sample that was included at least in triplo on each plate. Variation was determined over the glycopeptides with relative abundance >1% (O glycopeptide, total >89%; Asn144, total 100%; Asn340, total 100%) or >2% (truncated Asn340, total >92%).

	Plate	O-glycopeptide	Asn144	Asn340	Asn340 (truncated)
Intraplate	1	13.4%	10.8%	4.4%	16.1%
	2	17.9%	11.4%	8.9%	11.0%
Interplate		16.5%	11.3%	9.0%	18.7%

Supplementary Table S5 Mean and standard error of the mean (SEM) of all calculated glycosylation traits at all six time points. Abbreviations: trim = trimester; wkpp = weeks postpartum; GalNAc = *N*-acetylgalactosamine; Gal = galactose; SA = sialic acid (*N*-acetylneuraminic acid); % = percentage relative abundance; # = number calculated based on relative abundance.

		1st trim		2nd trim		3rd trim		6wkpp		12wkpp		>26wkpp	
		mean	SEM	mean	SEM	mean	SEM	mean	SEM	mean	SEM	mean	SEM
Asn 144	Sialylation (%)	61.39	0.96	62.89	0.83	63.12	0.90	59.08	1.05	59.23	0.98	59.07	0.80
	Bisection (%)	25.47	0.72	26.16	0.80	27.20	0.90	28.47	0.88	27.67	0.97	26.85	0.72
Asn 340	Sialylation (%)	95.18	0.17	94.99	0.22	95.34	0.16	95.40	0.19	95.31	0.23	95.22	0.25
	Bisection (%)	52.24	1.12	52.59	1.00	54.74	1.07	57.98	0.99	56.07	1.11	54.88	1.22
Asn340 (truncated)	Galactosylation (%)	99.84	0.01	99.84	0.01	99.83	0.01	99.82	0.01	99.85	0.01	99.84	0.01
	Sialylation (%)	89.56	0.21	89.28	0.28	89.12	0.19	89.26	0.23	89.21	0.27	89.25	0.27
	Fucosylation (%)	92.94	0.35	92.40	0.31	92.26	0.36	92.90	0.35	92.95	0.32	93.38	0.26
	Bisection (%)	51.76	1.04	52.67	1.03	53.87	1.00	58.45	1.03	56.86	1.19	55.36	0.94
	Triantennary (%)	5.46	0.26	6.36	0.41	6.10	0.28	5.40	0.29	5.60	0.30	5.24	0.25
O-glycosylation	GalNAc (#)	4.81	0.09	4.81	0.01	4.81	0.01	4.82	0.01	4.82	0.01	4.81	0.01
	Gal (#)	3.96	0.02	3.96	0.02	3.98	0.02	3.99	0.01	3.98	0.02	3.99	0.01
	SA (#)	3.03	0.03	3.03	0.04	3.02	0.03	3.04	0.02	3.08	0.03	3.05	0.03
	SA per Gal	0.77	0.01	0.77	0.01	0.76	0.01	0.76	0.01	0.77	0.01	0.77	0.01
	Gal per GalNAc	0.82	0.00	0.82	0.00	0.83	0.00	0.83	0.00	0.83	0.00	0.83	0.00
	SA>Gal (%)*	6.49	0.43	6.90	0.43	6.79	0.36	6.69	0.30	7.18	0.39	6.59	0.31
	GalNAc>Gal (%)†	61.36	0.94	64.37	0.87	60.27	0.86	59.99	0.76	60.72	0.79	59.81	0.79
	GalNAc>Gal (#)‡	0.85	0.02	0.85	0.02	0.83	0.02	0.82	0.01	0.83	0.01	0.82	0.01

* The percentage of *O*-glycopeptides with more sialic acids than galactoses.

† The percentage of *O*-glycopeptides with more GalNAcs than galactoses, indicative for at least one Tn-antigen.

‡ The number of GalNAcs more than galactoses; e.g. 1 * (relative abundance of H3N4) + 2 * (relative abundance of H2N4) etc.

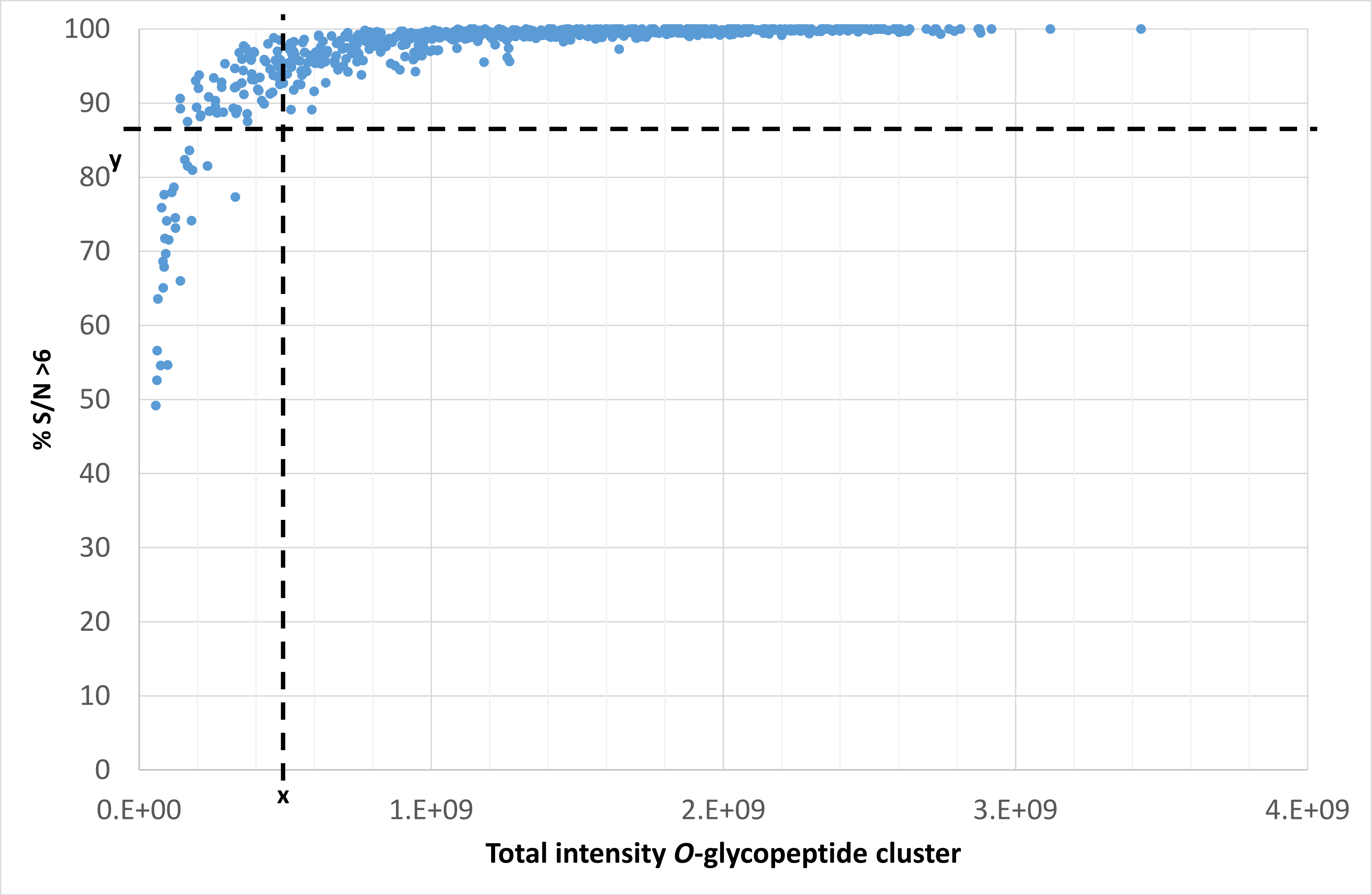

Supplementary Figure S1 Example of the plots used to determine cut-off values for glycopeptide cluster intensity (x-axis) and the relative abundance of analytes with signal-to-noise greater than 6 within that cluster (y-axis)

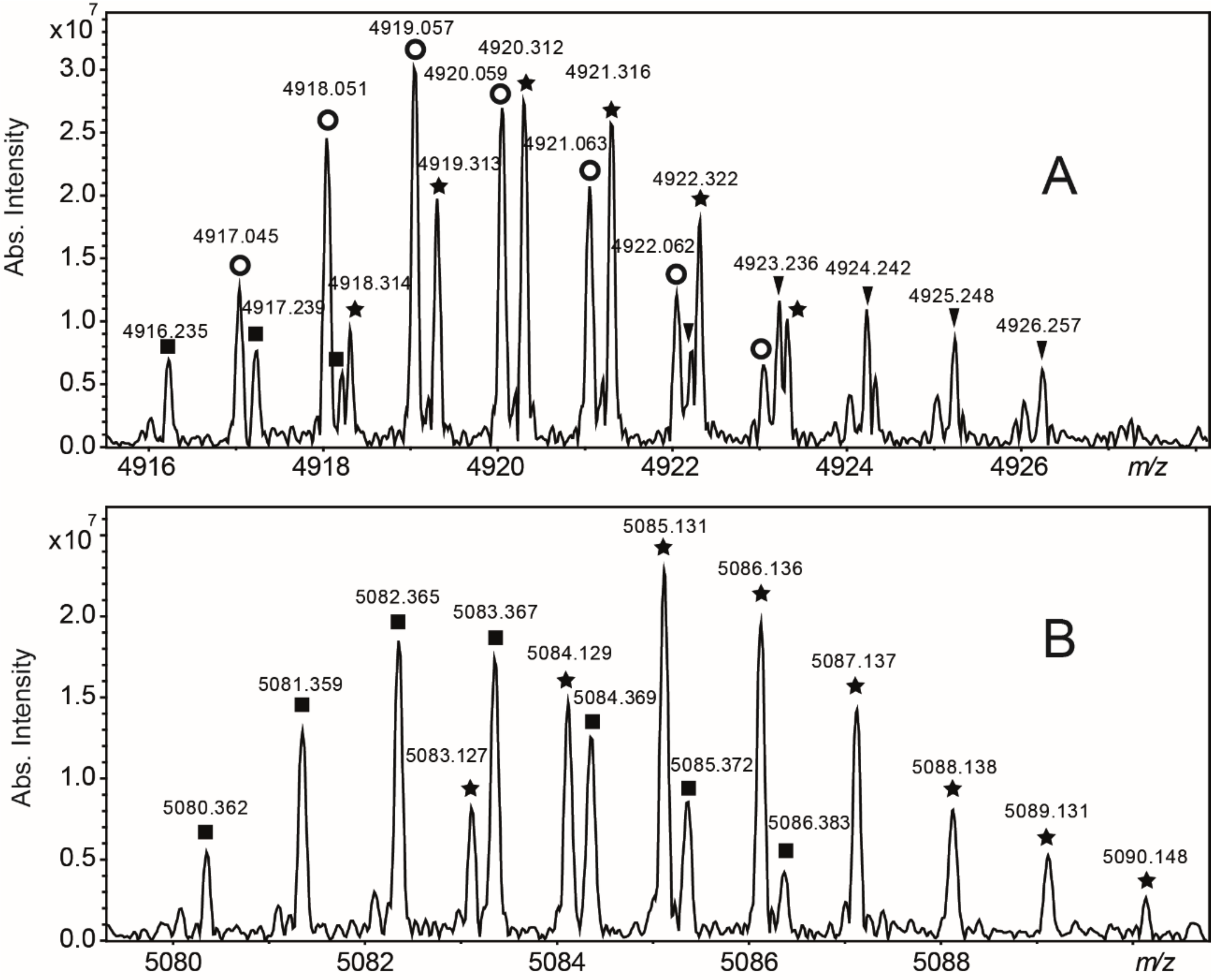

Supplementary Figure S2 Enlargements of MALDI-FTICR-MS spectra obtained from the analysis of *N*- and *O*-glycopeptides from serum IgA. The ultrahigh resolving power allowed both the resolution of isotopic distributions from species with close nominal masses and the accurate quantification of the selected peptides in all the spectra. Examples are of (**A**) H5N5F1S2 on the oxidized and truncated Asn340 glycopeptide (*m/z* 4917.047), and (**B**) H5N5S1 on Asn144 (*m/z* 5080.354).

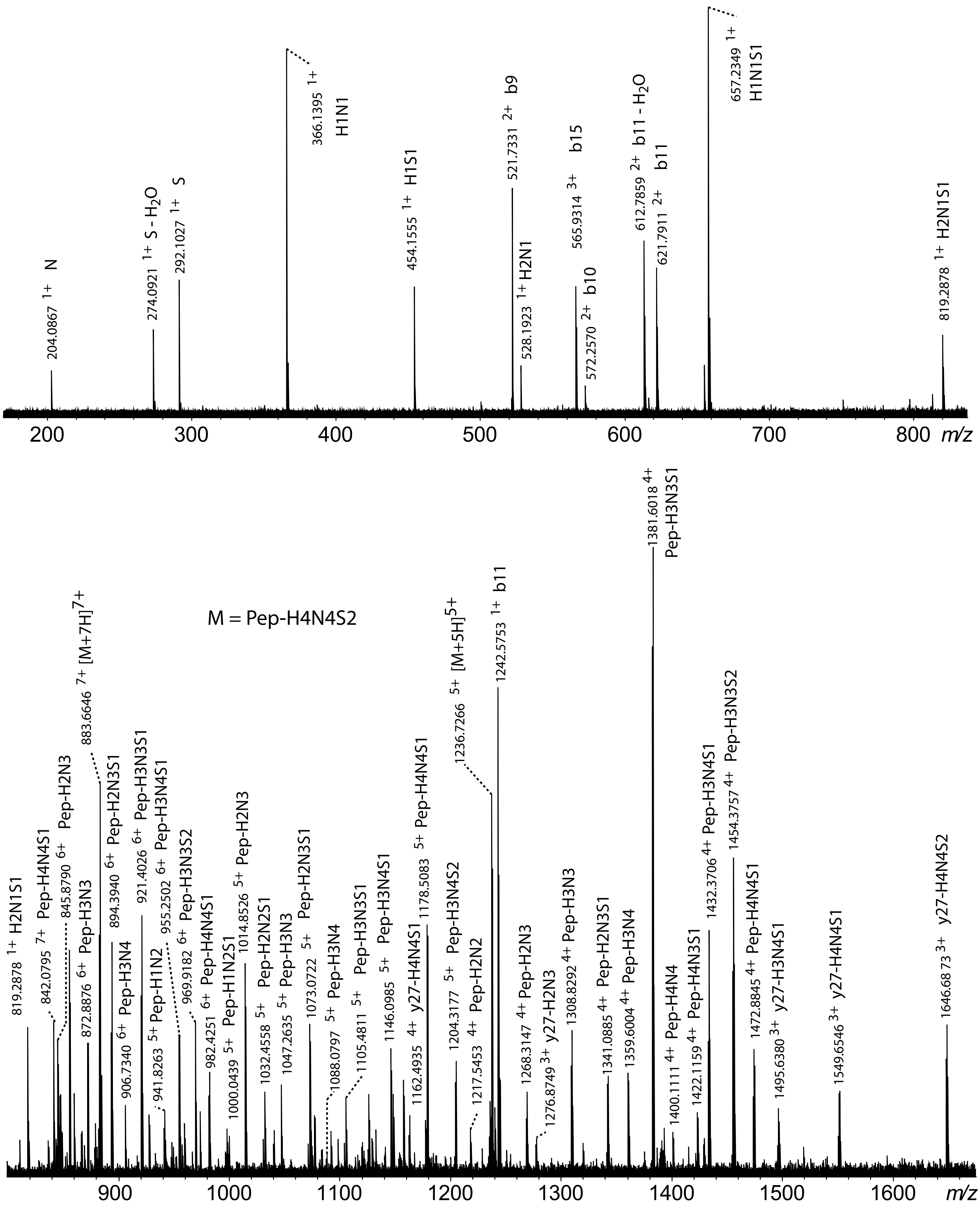

Supplementary Figure S3

MS/MS confirmation by ESI-FTICR-MS/MS of the IgA hinge region *O*-glycopeptide carrying H4N4S2.

Abbreviations: H = hexose; N = *N*-acetylhexosamine; S = *N*-acetylneuraminic acid; Pep = peptide moiety.

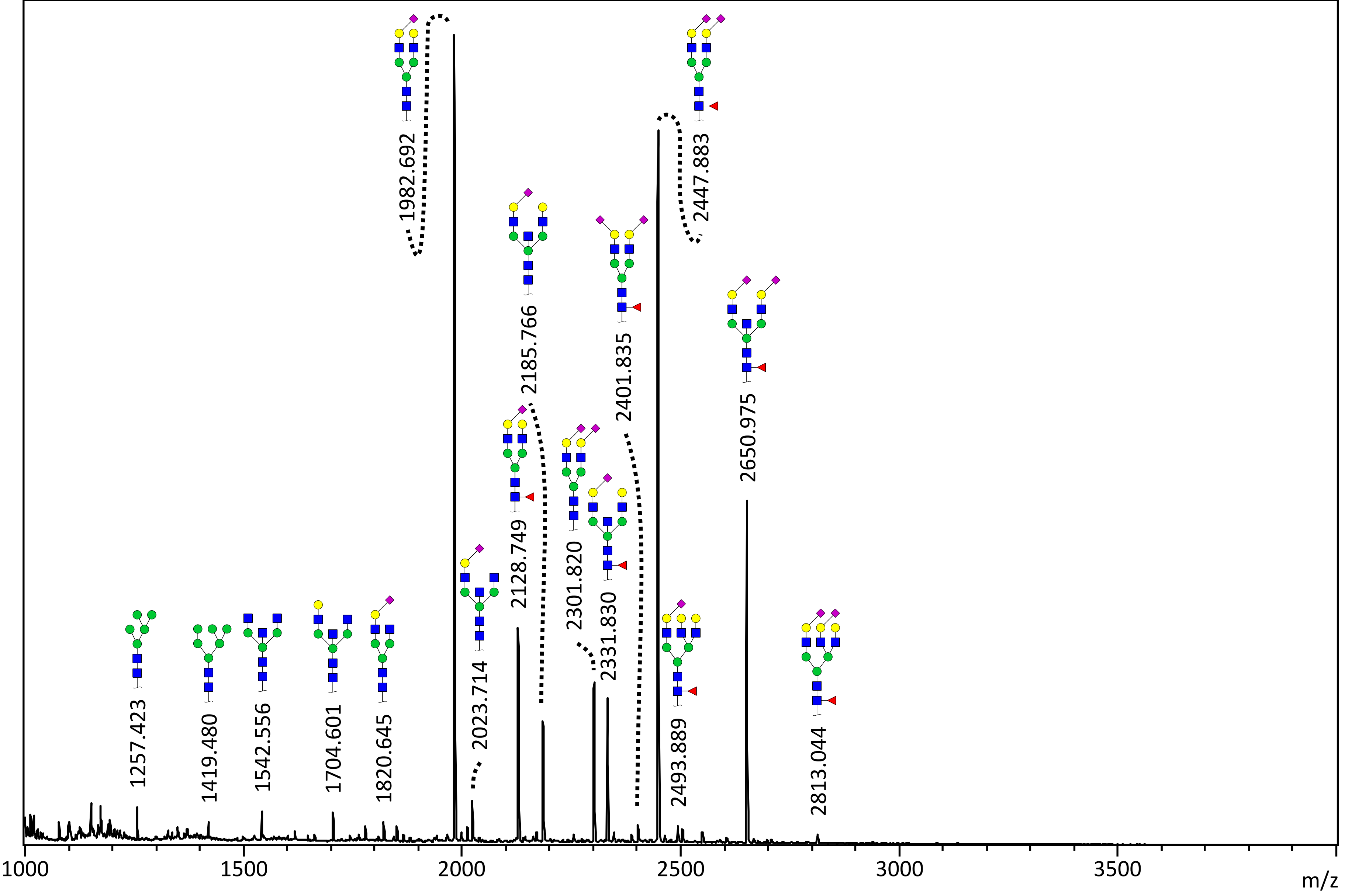

Supplementary Figure S4 Annotated spectrum of released and ethyl esterified IgA *N*-glycans. Of note, not all observed released *N*-glycans were found on the IgA1 *N*-glycopeptides; these could be derived from e.g. IgA2.

Supplementary Methods

MALDI-FTICR-MS settings

MALDI-FTICR-MS was performed with the smartbeam-II™ laser system at a frequency of 200 Hz. The 'medium' predefined shot pattern was used for the irradiation while the 'random walk' was allowed for a diameter of 600 μm. All MALDI-FTICR-MS spectra were acquired in the mass range from *m/z* 3499 to *m/z* 10000 as previously described with some modification.[1] Each mass spectrum was obtained from the sum of 15 scans of 150 laser shots each and using 256 K data points. The quadrupole mass filter was set to *m/z* 2500 while the time of flight to the ICR cell was 2.0 ms. Before detection, ions were trapped in the ICR cell using a back and front trapping voltage of 0.95 V and 0.8 V, respectively, while during detection, both voltages were set to 0.5 V. The required excitation power and pulse time were 34% and 15 μs, respectively.

ESI-FTICR-MS settings

ESI-FTICR-MS measurements were performed at an infusion rate of 2 μL/min using the quadrupole (Q) for precursor ion selection and a hexapole collision cell for collision-induced dissociation (CID). The ion funnels were operated at 100 and 6 V, respectively, with the skimmers at 15 V and 5 V. The trapping potentials were set at 1 V, the analyser entrance was maintained at −10 V, and side kick technology was used to further optimize peak shape and signal intensity. The required excitation power was 19% with a pulse time of 10 μs. MS/MS-experiments were performed by CID and fragment ion mass analysis in the ICR cell. For these experiments, the collision energy, the accumulation time in the hexapole collision cell and the isolation window in the Q were optimized for each precursor ion. Collision energies varied from -5.5 V to -10.5 V while the accumulation times varied from 5 s to 10 s.

LC-ESI-MS/MS

One microliter of sample was loaded onto a C18 μ-pre column (C18 PepMap 100, 300 μm x 5 mm, 5μm, 100 Å, Dionex/Thermo Scientific) with 10 μL/min of loading solvent (98% water/ 2% ACN/ 0.1% TFA) for 5 min. The analytes were then separated on a C18 analytical column (Acclaim PepMap RSLC, 75 μm × 15 cm, 5 μm, 2 μm, 100 Å, Dionex/Thermo Scientific). Elution was performed at a flow rate of 0.7 μL/min with solvent A (water containing 0.1% FA (v/v)) and solvent B (80% acetonitrile/ 20% water containing 0.1% FA (v/v)). A linear gradient of 3–50% solvent B in 42.5 min was applied followed by column washing and reconditioning.

Ionization using a captiveSpray was enhanced by a nanoBooster using acetonitrile with 0.2 bar. The source parameters were as followed: dry gas 3 L/min with 150˚C; capillary voltage 1200 V.

The mass spectrometer was tuned using ESI-L-low concentration tune mix (Agilent Technologies, Santa Clara, CA, USA). MS spectra were acquired within a mass range of 50-2800 *m/z* and a spectra rate of 1 Hz. MS transfer settings were as followed: Funnel 1RF 300 Vpp; Multipole RF 300 Vpp; Quadrupole ion energy 3 eV; low mass 100 m/z; collision cell Energy 5eV; pre pulse storage 10 μs. Basic stepping mode was applied for the collision RF (500-1300 Vpp), transfer time (90-130 μs) and MS/MS collision energy (80-140%) each 50% of the time. Detailed collision energies for quadruply charged ions, as were selected for MS/MS, were: *m/z* 500 at 20 eV, *m/z* 800 at 45 eV, *m/z* 1300 at 65 eV.

N-glycan release and sialic acid ethyl esterification

Eight replicates of the standard sample were reconstituted in 5 μL PBS, followed by the addition of 10 μL 2% SDS (w/v). The samples were incubated for 10 min on a multiwell plate shaker before a 30 minute

incubation at 60°C. Subsequently, 10 μL of a 1:1 solution of 4% NP-40 and 5X PBS containing 1U PNGaseF was added. The glycan release was performed overnight at 37°C.

The released glycans were subjected to a sialic acid stabilization step as described before [2,3], resulting in linkage specific modification of the sialic acids. Briefly, 10 μL released glycans were added to 100 μL 250 mM EDC (1-ethyl-3-(3-dimethylaminopropyl)carbodiimide hydrochloride; Fluorochem, Hadfield, UK) /250 mM HOBt (hydroxybenzotriazole hydrate; Sigma Aldrich) and incubated for 1h at 37°C. After the incubation, 100 μL ACN was added, followed by a 15 min incubation at -20°C. The samples were allowed to warm up to ambient temperature, after which the glycans were purified by cotton HILIC-SPE, as described before [3]. Purified glycans were spotted on a Bruker AnchorChip plate (part number 209514, 800 μm anchor; Bruker Daltonics) and mixed on spot with 5 mg/mL Super-DHB (Sigma) containing 1 mM NaOH. The sample were measured in reflectron positive mode on a Bruker fleXtreme MALDI-TOF mass spectrometer.

LC-ion trap-MS/MS for protein and peptide identification

IgA sample purity was assessed by proteomics analysis. The sample was prepared as described in the main manuscript. One microliter digest was diluted 100 times before injection onto the LC-ion trap MS/MS system. The analysis was performed as described before.[4] Using Data Analysis 4.0 (Bruker Daltonics, Bremen, Germany) compounds were generated from the LC-MS/MS runs, and the data was exported in the Mascot Generic File format. Mascot Deamon 2.2.2 allowed batch processing to indentify peptides using the uniprothuman 20150204 database with the addition of the truncated IGHA1, referred to as IGHA1b (89797 sequences; 35657535 residues), and the Mascot algorithm (Mascot 2.5.1, Matrix Science, London, UK). Mass tolerance was set at ±0.5 Da for both peptide and MS/MS fragments. The number of ^{13}C was set to 1. Oxidation of methionine (variable), deamidation of asparagine/glutamine

(variable), and carbamidomethylation of cysteine (fixed) were selected as modification. Positive protein identification required at least two significant sequences and peptides with a score above 30.

In addition, a sample was digested O/N with PNGaseF to release the N-glycans. After the digestion, the sample and data were treated as described above.

The resulting data is summarized in Supplemental Table S1.

Formulae to calculate the different glycosylation traits.

The following formulae were used to calculate the glycosylation traits for both *N*- and *O*- glycosylation of IgA1. For earlier convenience all relative abundances were multiplied by 100. Therefore, the calculated traits resulting in the number of GalNAcs, galactoses or sialic acids were divided by 100. Abbreviations: Q = *O*-glycopeptide; H = hexose; N = *N*-acetylhexosamine; F = fucose; S = sialic acid; R = Asn144 containing *N*-glycopeptide; T = Asn340 containing *N*-glycopeptide; U = truncated Asn340 containing *N*-glycopeptide; _O: oxidized.

Number of *N*-acetylgalactosamines: (3*(Q1H2N3F0S1 + Q1H2N3F0S2 + Q1H3N3F0S1 + Q1H3N3F0S2 + Q1H3N3F0S3)+ 4*(Q1H2N4F0S0 + Q1H2N4F0S1 + Q1H2N4F0S2 + Q1H3N4F0S0 + Q1H3N4F0S1 + Q1H3N4F0S2 + Q1H3N4F0S3 + Q1H3N4F0S4 + Q1H4N4F0S0 + Q1H4N4F0S1 + Q1H4N4F0S2 + Q1H4N4F0S3 + Q1H4N4F0S4 + Q1H4N4F0S5 + Q1H4N4F0S6) + 5*(Q1H2N5F0S1 + Q1H2N5F0S2 + Q1H3N5F0S0 + Q1H3N5F0S1 + Q1H3N5F0S2 + Q1H3N5F0S3 + Q1H3N5F0S4 + Q1H4N5F0S0 + Q1H4N5F0S1 + Q1H4N5F0S2 + Q1H4N5F0S3 + Q1H4N5F0S4 + Q1H4N5F0S5 + Q1H4N5F0S6 + Q1H5N5F0S1 + Q1H5N5F0S2 + Q1H5N5F0S3 + Q1H5N5F0S4 + Q1H5N5F0S5 + Q1H5N5F0S6 + Q1H5N5F0S7) + 6*(Q1H3N6F0S2 + Q1H3N6F0S3 + Q1H4N6F0S1 + Q1H4N6F0S2 + Q1H4N6F0S3 + Q1H4N6F0S4 + Q1H4N6F0S5 + Q1H5N6F0S2 + Q1H5N6F0S3 + Q1H5N6F0S4 + Q1H5N6F0S5 + Q1H6N6F0S4 + Q1H6N6F0S5))/100

Number of galactoses: (2*(Q1H2N3F0S1 + Q1H2N3F0S2 + Q1H2N4F0S0 + Q1H2N4F0S1 + Q1H2N4F0S2 + Q1H2N5F0S1 + Q1H2N5F0S2) + 3*(Q1H3N3F0S1 + Q1H3N3F0S2 + Q1H3N3F0S3 + Q1H3N4F0S0 + Q1H3N4F0S1 + Q1H3N4F0S2 + Q1H3N4F0S3 + Q1H3N4F0S4 + Q1H3N5F0S0 + Q1H3N5F0S1 + Q1H3N5F0S2 + Q1H3N5F0S3 + Q1H3N5F0S4 + Q1H3N6F0S2 + Q1H3N6F0S3) + 4*(Q1H4N4F0S0 + Q1H4N4F0S1 + Q1H4N4F0S2 + Q1H4N4F0S3 + Q1H4N4F0S4 + Q1H4N4F0S5 + Q1H4N4F0S6 + Q1H4N5F0S0 + Q1H4N5F0S1 + Q1H4N5F0S2 + Q1H4N5F0S3 + Q1H4N5F0S4 + Q1H4N5F0S5 + Q1H4N5F0S6 + Q1H4N6F0S1 + Q1H4N6F0S2 + Q1H4N6F0S3 + Q1H4N6F0S4 + Q1H4N6F0S5) + 5*(Q1H5N5F0S1 + Q1H5N5F0S2 + Q1H5N5F0S3 + Q1H5N5F0S4 + Q1H5N5F0S5 + Q1H5N5F0S6 + Q1H5N5F0S7 + Q1H5N6F0S2 + Q1H5N6F0S3 + Q1H5N6F0S4 + Q1H5N6F0S5) + 6*(Q1H6N6F0S4 + Q1H6N6F0S5))/100

Number of sialic acids: (1*(Q1H2N3F0S1 + Q1H2N4F0S1 + Q1H2N5F0S1 + Q1H3N3F0S1 + Q1H3N4F0S1 + Q1H3N5F0S1 + Q1H4N4F0S1 + Q1H4N5F0S1 + Q1H4N6F0S1 + Q1H5N5F0S1) + 2*(Q1H2N3F0S2 + Q1H2N4F0S2 + Q1H2N5F0S2 + Q1H3N3F0S2 + Q1H3N4F0S2 + Q1H3N5F0S2 + Q1H3N6F0S2 + Q1H4N4F0S2 + Q1H4N5F0S2 + Q1H4N6F0S2 + Q1H5N5F0S2 + Q1H5N6F0S2) + 3*(Q1H3N3F0S3 + Q1H3N4F0S3 + Q1H3N5F0S3 + Q1H3N6F0S3 + Q1H4N4F0S3 + Q1H4N5F0S3 + Q1H4N6F0S3 + Q1H5N5F0S3 + Q1H5N6F0S3) + 4*(Q1H3N4F0S4 + Q1H3N5F0S4 + Q1H4N4F0S4 + Q1H4N5F0S4 + Q1H4N6F0S4 + Q1H5N5F0S4 + Q1H5N6F0S4 + Q1H6N6F0S4) + 5*(Q1H4N4F0S5 + Q1H4N5F0S5 + Q1H4N6F0S5 + Q1H5N5F0S5 + Q1H5N6F0S5 + Q1H6N6F0S5) + 6*(Q1H4N4F0S6 + Q1H4N5F0S6 + Q1H5N5F0S6) + 7*(Q1H5N5F0S7))/100

Ratio of sialic acids per galactose: ‘Number of sialic acids’ / ‘Number of galactoses’

Ratio of galactoses per GalNAc: ‘Number of galactoses‘ / ‘Number of GalNAcs’

Abundance of peptides with more GalNAc then galactoses: Q1H2N3F0S1 + Q1H2N3F0S2 + Q1H2N4F0S0 + Q1H2N4F0S1 + Q1H2N4F0S2 + Q1H2N5F0S1 + Q1H2N5F0S2 + Q1H3N4F0S0 + Q1H3N4F0S1 +

Q1H3N4F0S2 + Q1H3N4F0S3 + Q1H3N4F0S4 + Q1H3N5F0S0 + Q1H3N5F0S1 + Q1H3N5F0S2 + Q1H3N5F0S3 + Q1H3N5F0S4 + Q1H3N6F0S2 + Q1H3N6F0S3 + Q1H4N5F0S0 + Q1H4N5F0S1 + Q1H4N5F0S2 + Q1H4N5F0S3 + Q1H4N5F0S4 + Q1H4N5F0S5 + Q1H4N5F0S6 + Q1H4N6F0S1 + Q1H4N6F0S2 + Q1H4N6F0S3 + Q1H4N6F0S4 + Q1H4N6F0S5 + Q1H5N6F0S2 + Q1H5N6F0S3 + Q1H5N6F0S4 + Q1H5N6F0S5

Abundance of peptides with more sialic acids then galactoses: Q1H3N4F0S4 + Q1H3N5F0S4 + Q1H4N4F0S5 + Q1H4N4F0S6 + Q1H4N5F0S5 + Q1H4N5F0S6 + Q1H4N6F0S5 + Q1H5N5F0S6 + Q1H5N5F0S7

Asn144 sialylation: 0.5*(R1H5N4F0S1 + R1H5N5F0S1) + 1*(R1H5N4F0S2 + R1H5N5F0S2)

Asn144 bisection: R1H5N5F0S0 + R1H5N5F0S1 + R1H5N5F0S2

Asn340 sialylation: 0.5*(T1H5N4F1S1_O1) + 1*(T1H5N4F1S2_O1 + T1H5N5F1S2_O1)

Asn340 bisection: T1H5N5F1S2_O1

Truncated Asn340 diantennary galactosylation: ((0.5*(U1H4N4F0S1) + 1*(U1H5N4F0S1 + U1H5N4F0S1_O1 + U1H5N4F0S2_O1 + U1H5N4F1S0_O1 + U1H5N4F1S1 + U1H5N4F1S1_O1 + U1H5N4F1S2 + U1H5N4F1S2_O1 + U1H5N5F0S1 + U1H5N5F0S1_O1 + U1H5N5F0S2 + U1H5N5F1S0_O1 + U1H5N5F1S1 + U1H5N5F1S1_O1 + U1H5N5F1S2 + U1H5N5F1S2_O1))/(U1H4N4F0S1 + U1H5N4F0S1 + U1H5N4F0S1_O1 + U1H5N4F0S2_O1 + U1H5N4F1S0_O1 + U1H5N4F1S1 + U1H5N4F1S1_O1 + U1H5N4F1S2 + U1H5N4F1S2_O1 + U1H5N5F0S1 + U1H5N5F0S1_O1 + U1H5N5F0S2 + U1H5N5F1S0_O1 + U1H5N5F1S1 + U1H5N5F1S1_O1 + U1H5N5F1S2 + U1H5N5F1S2_O1))*100

Truncated Asn340 diantennary sialylation: ((0.5*(U1H4N4F0S1 + U1H5N4F0S1 + U1H5N4F0S1_O1 + U1H5N4F1S1 + U1H5N4F1S1_O1 + U1H5N5F0S1 + U1H5N5F0S1_O1 + U1H5N5F1S1 + U1H5N5F1S1_O1) + 1*(U1H5N4F0S2_O1 + U1H5N4F1S2 + U1H5N4F1S2_O1 + U1H5N5F0S2 + U1H5N5F1S2 +

U1H5N5F1S2_O1))/(U1H4N4F0S1 + U1H5N4F0S1 + U1H5N4F0S1_O1 + U1H5N4F0S2_O1 + U1H5N4F1S0_O1 + U1H5N4F1S1 + U1H5N4F1S1_O1 + U1H5N4F1S2 + U1H5N4F1S2_O1 + U1H5N5F0S1 + U1H5N5F0S1_O1 + U1H5N5F0S2 + U1H5N5F1S0_O1 + U1H5N5F1S1 + U1H5N5F1S1_O1 + U1H5N5F1S2 + U1H5N5F1S2_O1))*100

Truncated Asn340 diantennary fucosylation: ((U1H5N4F1S0_O1 + U1H5N4F1S1 + U1H5N4F1S1_O1 + U1H5N4F1S2 + U1H5N4F1S2_O1 + U1H5N5F1S0_O1 + U1H5N5F1S1 + U1H5N5F1S1_O1 + U1H5N5F1S2 + U1H5N5F1S2_O1)/(U1H4N4F0S1 + U1H5N4F0S1 + U1H5N4F0S1_O1 + U1H5N4F0S2_O1 + U1H5N4F1S0_O1 + U1H5N4F1S1 + U1H5N4F1S1_O1 + U1H5N4F1S2 + U1H5N4F1S2_O1 + U1H5N5F0S1 + U1H5N5F0S1_O1 + U1H5N5F0S2 + U1H5N5F1S0_O1 + U1H5N5F1S1 + U1H5N5F1S1_O1 + U1H5N5F1S2 + U1H5N5F1S2_O1))*100

Truncated Asn340 diantennary bisection: ((U1H5N5F0S1 + U1H5N5F0S1_O1 + U1H5N5F0S2 + U1H5N5F1S0_O1 + U1H5N5F1S1 + U1H5N5F1S1_O1 + U1H5N5F1S2 + U1H5N5F1S2_O1)/(U1H4N4F0S1 + U1H5N4F0S1 + U1H5N4F0S1_O1 + U1H5N4F0S2_O1 + U1H5N4F1S0_O1 + U1H5N4F1S1 + U1H5N4F1S1_O1 + U1H5N4F1S2 + U1H5N4F1S2_O1 + U1H5N5F0S1 + U1H5N5F0S1_O1 + U1H5N5F0S2 + U1H5N5F1S0_O1 + U1H5N5F1S1 + U1H5N5F1S1_O1 + U1H5N5F1S2 + U1H5N5F1S2_O1))*100

Truncated Asn340 triantennary: U1H6N5F1S1_O1 + U1H6N5F1S3_O1 + U1H6N6F0S0_O1

References

1 Nicolardi, S., van der Burgt, Y. E. M., Wuhrer, M. & Deelder, A. M. Mapping O-glycosylation of apolipoprotein C-III in MALDI-FT-ICR protein profiles. *Proteomics* **13**, 992-1001, doi:10.1002/pmic.201200293 (2013).

2 Reiding, K. R., Blank, D., Kuijper, D. M., Deelder, A. M. & Wuhrer, M. High-Throughput Profiling of Protein N-Glycosylation by MALDI-TOF-MS Employing Linkage-Specific Sialic Acid Esterification. *Anal. Chem.* **86**, 5784-5793, doi:Doi 10.1021/Ac500335t (2014).

3 Bondt, A. *et al.* Immunoglobulin G (IgG) Fab glycosylation analysis using a new mass spectrometric high-throughput profiling method reveals pregnancy-associated changes. *Mol. Cell. Proteomics* **13**, 3029-3039, doi:10.1074/mcp.M114.039537 (2014).

4 Zauner, G. *et al.* Glycoproteomic Analysis of Human Fibrinogen Reveals Novel Regions of O-Glycosylation. *J. Proteome Res.* **11**, 5804-5814, doi:10.1021/pr3005937 (2012).
